# Class II phosphatidylinositol 3-kinase-C2α is essential for Notch signaling by regulating the endocytosis of γ-secretase in endothelial cells

**DOI:** 10.1038/s41598-021-84548-4

**Published:** 2021-03-04

**Authors:** Shota Shimizu, Kazuaki Yoshioka, Sho Aki, Yoh Takuwa

**Affiliations:** Department of Physiology, Kanazawa University School of Medicine, 13-1 Takara-machi, Kanazawa, Ishikawa 920-8640 Japan

**Keywords:** Biochemistry, Cell biology, Developmental biology, Physiology, Cardiology, Molecular medicine

## Abstract

The class II α-isoform of phosphatidylinositol 3-kinase (PI3K-C2α) plays a crucial role in angiogenesis at least in part through participating in endocytosis and, thereby, endosomal signaling of several cell surface receptors including VEGF receptor-2 and TGFβ receptor in vascular endothelial cells (ECs). The Notch signaling cascade regulates many cellular processes including cell proliferation, cell fate specification and differentiation. In the present study, we explored a role of PI3K-C2α in Delta-like 4 (Dll4)-induced Notch signaling in ECs. We found that knockdown of PI3K-C2α inhibited Dll4-induced generation of the signaling molecule Notch intracellular domain 1 (NICD1) and the expression of Notch1 target genes including *HEY1*, *HEY2* and *NOTCH3* in ECs but not in vascular smooth muscle cells. PI3K-C2α knockdown did not inhibit Dll4-induced endocytosis of cell surface Notch1. In contrast, PI3K-C2α knockdown as well as clathrin heavy chain knockdown impaired endocytosis of Notch1-cleaving protease, γ-secretase complex, with the accumulation of Notch1 at the perinuclear endolysosomes. Pharmacological blockage of γ-secretase also induced the intracellular accumulation of Notch1. Taken together, we conclude that PI3K-C2α is required for the clathrin-mediated endocytosis of γ-secretase complex, which allows for the cleavage of endocytosed Notch1 by γ-secretase complex at the endolysosomes to generate NICD1 in ECs.

## Introduction

Phosphatidylinositol 3-kinases (PI3Ks) are a family of lipid kinases which phosphorylate membrane phosphoinositides at the 3′-position of their inositol rings. The resulting lipid products, phosphatidylinositol 3-phosphate (PtdIns(3)P), phosphatidylinositol 3,4-bisphosphate (PtdIns(3,4)P_2_) and phosphatidylinositol 3,4,5-trisphosphate (PtdIns(3,4,5)P_3_), have roles in the regulation of a large set of biological processes^1,2^. PI3Ks have eight isoforms, which are divided into three classes based on structural features and lipid substrate preferences. Class I PI3Ks consist of four catalytic subunits, p110α, p110β, p110γ, and p110δ, and have been most extensively studied. They are activated by tyrosine kinases and G-protein coupled receptors to convert the PtdIns(4,5)P_2_ into PtdIns(3,4,5)P_3_, which acts as a second messenger to bind and modulate the activity of pleckstrin homology (PH) domain-containing effectors including protein kinase-B (also known as Akt) and guanine nucleotide exchange factors for Rac, stimulating cell proliferation and migration^1,3^. Vps34, the unique member of Class III PI3K, generates PtdIns(3)P to promote autophagosome formation ^1,4^.

Class II PI3Ks, which comprise PI3K-C2α, PI3K-C2β and PI3K-C2γ, are thought to generate PtdIns(3,4)P_2_ and PtdIns(3)P on the plasma membrane and the endosomal compartments, regulating vesicular trafficking, which includes receptor endocytosis^5–9^, endosomal trafficking of glucose transporters^10^, neurosecretory granule release^11^ and insulin secretion^12^. Previous in vitro studies^5,9,13,14^ have demonstrated that PI3K-C2α is distinct from other PI3Ks in terms of the presence of the clathrin-binding domain at its N-terminus and localized at the plasma membrane, clathrin-coated pits and vesicles, endosomes, *trans*-Golgi network and mitotic spindles. We have reported that either global- or endothelial-specific deletion of PI3K-C2α gene in mice resulted in embryonic lethality due to severe defects in angiogenesis and that PI3K-C2α also plays a crucial role in the maintenance of vascular barrier integrity in adult mice^5^. More recent studies highlighted its role in the clathrin-mediated endocytosis and generation of primary cilium^9,15^. We have shown as the mechanisms underlying defective angiogenesis that PI3K-C2α is required for ligand-induced endocytosis of vascular endothelial growth factor (VEGF) receptor-2 (VEGFR2)^5^, sphingosine 1-phosphate receptor-1 (S1P_1_)^6^ and transforming growth factor β (TGFβ) receptor^7^ in vascular endothelial cells (ECs), and subsequent endosomal signaling including the activation of Rho, Rac and Smads. However, the molecular mechanisms of how PI3K-C2α is involved in vascular formation are not fully understood.

The Notch signaling pathway is evolutionary conserved from flies to mammals and essential for cell-fate decision and tissue development including vascular formation^16–18^. Mammals have four Notch paralogues (Notch1-4) and their five ligands including Delta-like (Dll1, Dll3, Dll4) and Jagged (Jag1, Jag2). Genetic alterations of Notch components in ECs resulted in disorganized and nonfunctional vasculatures due to pathological angiogenesis and impaired vascular remodeling, leading to embryonic lethality^19,20^. Dll4-induced Notch signaling pathway plays a pivotal role in tip-cell *versus* stalk-cell selection in sprouting angiogenesis^21–24^. Notch receptors are large type-I transmembrane proteins, which are present at the cell surface as heterodimers composed of extracellular domain and transmembrane-intracellular domain (TM-IC) after glycosylation and cleavage by Furin-like convertases (S1 cleavage) in the Golgi^25^. Notch signaling is initiated by the binding of a ligand presented on neighboring cells, which leads to the conformational change of Notch and its proteolytic cleavage by a disintegrin and metalloproteinase (ADAM) at juxtamembrane site (S2 cleavage)^26,27^. The resulting truncated form of Notch (Notch extracellular truncation (NEXT)) is eventually cleaved by the γ-secretase (S3 cleavage) within the transmembrane domain to release the Notch intracellular domain (NICD)^28,29^, which translocates to the nucleus and regulates transcription of the target genes. Because there are no second messengers downstream of NICD, the regulation of NICD production is crucial to fine-tune the signal intensity. NICD production is precisely controlled by membrane trafficking^30,31^. Genetic studies in flies revealed that endocytosis of Notch is critical for proper NICD production^32,33^. Upon ligand binding, Notch is cleaved by ADAM at the plasma membrane^34,35^ or at endosomes after dynamin-dependent endocytosis^36,37^. Finally, the resultant NEXT is cleaved by γ-secretase at endosomes, leading to the generation of NICD^36,37^. It is demonstrated that γ-secretase is constitutively internalized through clathrin-dependent endocytosis^38^. In contrast, some previous studies suggest that the endocytosis is not essential for γ-secretase-mediated cleavage of Notch^39,40^. Thus, further studies are needed to fully understand the role of endocytosis in Notch signaling pathway.

In the present study, we explored possible involvement of PI3K-C2α-mediated endocytosis in Notch signaling in ECs. We found that Dll4- and Jag1-induced NICD1 production and its target gene expression were dependent on PI3K-C2α in ECs, but not in vascular smooth muscle cells (SMCs). Knockdown of PI3K-C2α as well as clathrin heavy chain (CHC), inhibited the internalization of γ-secretase complex from the cell surface and resulted in the accumulation of Notch1 at the endolysosomal compartment, suggesting that PI3K-C2α is involved in clathrin-dependent endocytosis of γ-secretase complex and subsequent Notch1 cleavage by γ-secretase complex at the endolysosomal compartments. Taken together, these observations show that PI3K-C2α is required for Notch signaling in ECs through the involvement in clathrin-mediated endocytosis of γ-secretase complex.

## Results

### Class II PI3K-C2α is required for ligand-induced Notch1 signaling in vascular ECs but not SMCs

**Figure 1:**
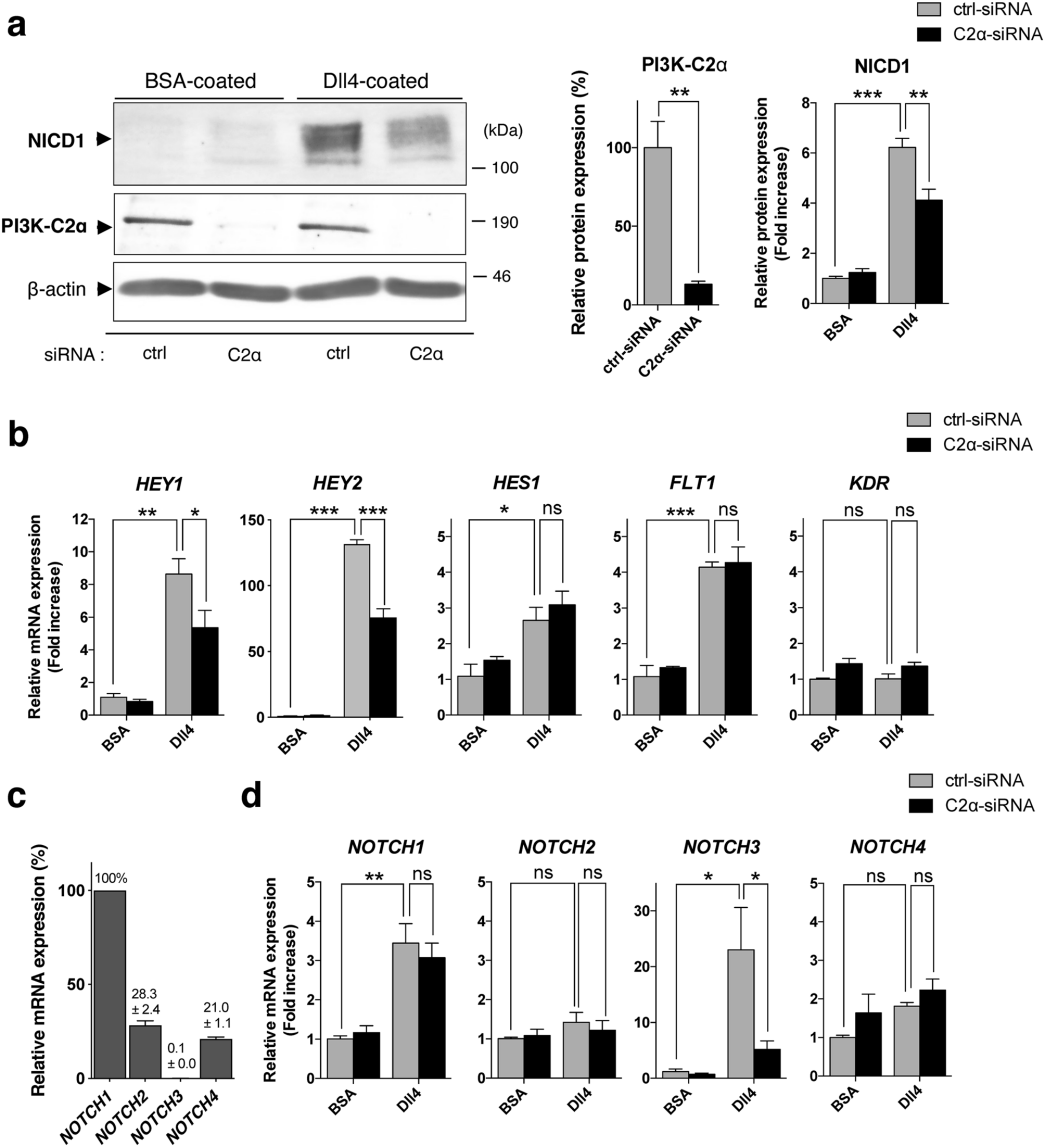
PI3K-C2α is required for Dll4-induced Notch signaling in HUVECs. (**a**) Representative Western blot images showing the effects of PI3K-C2α knockdown on Dll4-induced NICD1 production. PI3K-C2α-siRNA- or control (ctrl)-siRNA-transfected HUVECs were incubated on Dll4- or BSA-coated plates and 24 h later underwent Western blot analysis using anti-NICD1 and anti-PI3K-C2α antibodies. Right two panels show the quantified data of PI3K-C2α and NICD1 protein levels (n = 3). Statistical significance was assessed with two-tailed unpaired Student’s* t* test for PI3K-C2α and two-way ANOVA followed by Bonfferroni’s post-hoc test for NICD1. (**b**) The effects of PI3K-C2α knockdown on Dll4-induced Notch target gene expression. PI3K-C2α-siRNA- or ctrl-siRNA-transfected HUVECs were incubated on Dll4- or BSA-coated plates and 24 h later subjected to qPCR analysis (n = 5). Statistical significance was assessed with two-way ANOVA followed by Bonfferroni’s post-hoc test. (**c**) Relative mRNA expression levels of Notch paralogues compared with *NOTCH1*. The values on the top of bars represent the means ± SEM. HUVECs were cultured on BSA-coated plates for 24 h and underwent qPCR analysis (n = 3). (**d**) The effects of PI3K-C2α knockdown on mRNA expression of Notch paralogues. PI3K-C2α-siRNA- or ctrl-siRNA-transfected HUVECs were incubated on Dll4- or BSA-coated plates and 24 h later subjected to qPCR analysis (n = 3). In a-d, data are presented as means ± SEM from three to five independent experiments. Statistical significance was presented as *P < 0.05, **P < 0.01 or ***P < 0.001, respectively. ns: not significant.

**Figure 2:**
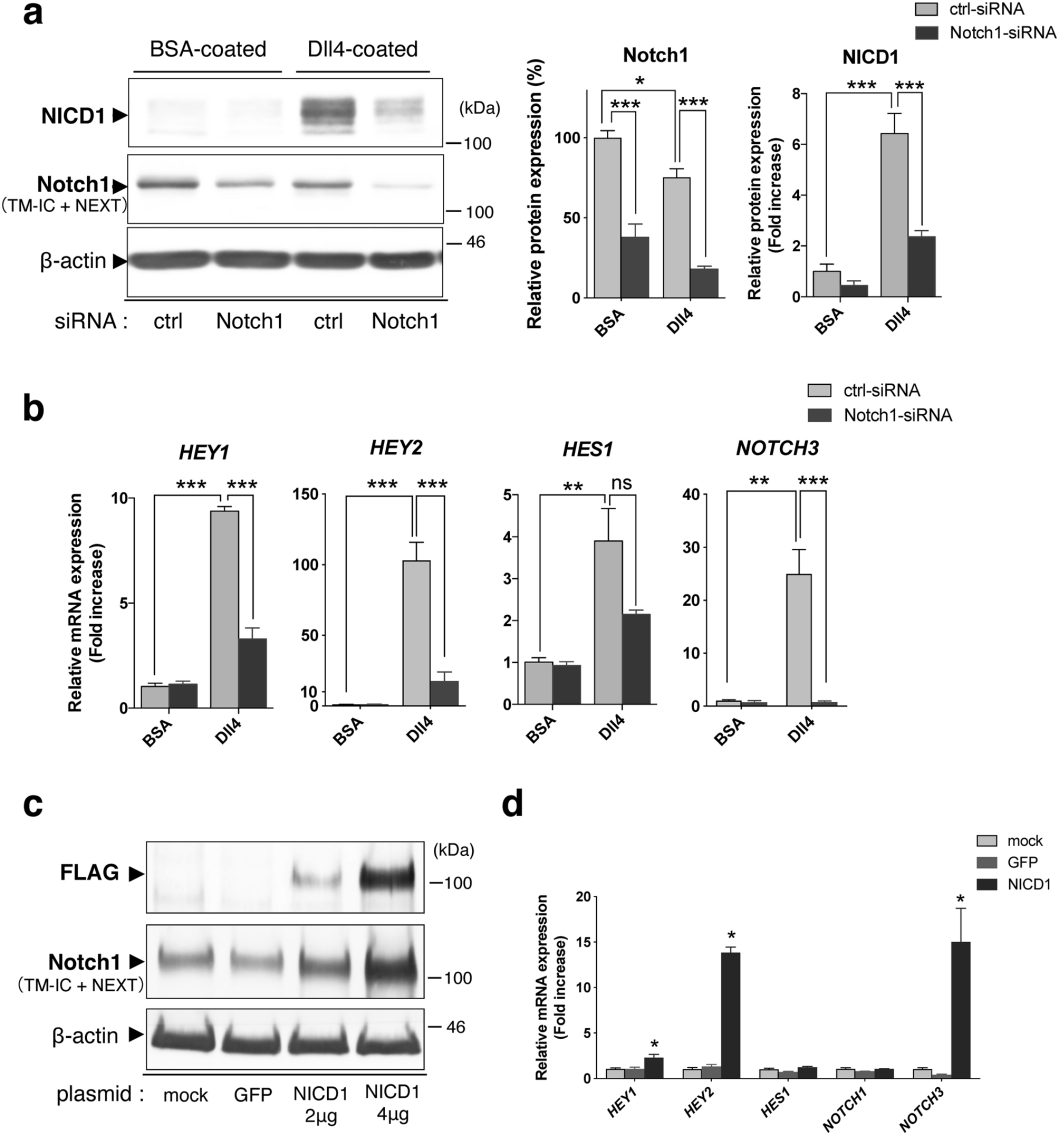
*HEY1, HEY2* and *NOTCH3* are Notch1 target genes in HUVECs. (**a**) The effects of Notch1 knockdown on Dll4-induced NICD1 production in HUVECs. Notch1-siRNA- or ctrl-siRNA-transfected cells were incubated on Dll4- or BSA-coated plates and 24 h later subjected to Western blot analysis for Notch1 and NICD1. β-actin was analyzed as a loading control. Right two panels show the quantified data of Notch1 (TM-IC + NEXT) and NICD1 protein levels (n = 3). Statistical significance was assessed with two-way ANOVA followed by Bonfferroni’s post-hoc test. TM-IC: transmembrane and intracellular domain of full-length Notch1. NEXT: Notch1 extracellular truncation. (**b**) The effects of Notch1 knockdown on Dll4-induced Notch target gene expression. Notch1-siRNA- or ctrl-siRNA-transfected HUVECs were incubated on Dll4- or BSA-coated plates and 24 h later underwent qPCR analysis (n = 3). Statistical significance was assessed with two-way ANOVA followed by Bonfferroni’s post-hoc test. (**c**) Expression of 3xFLAG-tagged-NICD1 in HUVECs. Cells were transfected with the indicated expression vectors or not, and 24 h later harvested for Western blot analysis using anti-FLAG, anti-Notch1, and anti-β-actin antibodies. GFP denotes transfection with the GFP expression plasmid (4 μg) as a control. (**d**) The effects of NICD1 overexpression on Notch target gene expression in HUVECs. Cells were transfected with FLAG-tagged-NICD1 expression plasmid (4 μg) or GFP expression plasmid (4 μg) or mock-transfected, and 24 h later harvested for qPCR analysis (n = 3). Statistical significance between mock-treated cells and NICD1-overexpressing cells was assessed with one-way ANOVA followed by Bonfferroni’s post-hoc test. Statistical significance was presented as *P < 0.05, **P < 0.01 or ***P < 0.001, respectively. ns: not significant.

**Figure 3:**
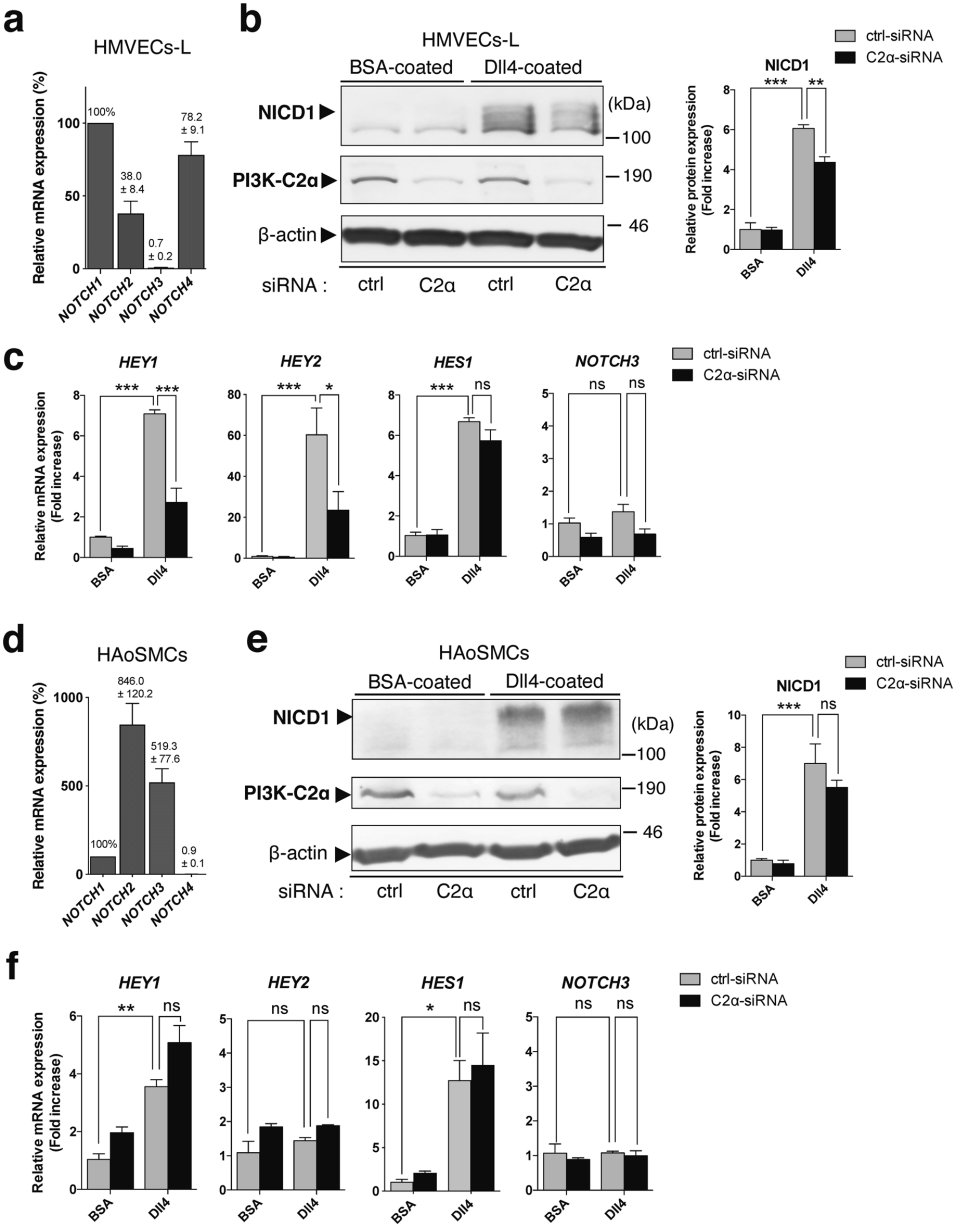
PI3K-C2α is required for Dll4-induced Notch signaling in endothelial cells but not in smooth muscle cells. (**a**, **d**) Relative mRNA expression level of Notch paralogues compared with *NOTCH1*. The values on the top of bars represent the means ± SEM. Isolated total RNAs of HMVECs-L (**a**) and HAoSMCs (**d**) were subjected to qPCR analysis, respectively. (n = 3). (**b**, **e**) The effects of PI3K-C2α knockdown on Dll4-induced NICD1 production in HMVECs-L (**b**) and HAoSMCs (**e**). PI3K-C2α-siRNA- or ctrl-siRNA-transfected cells were incubated on Dll4- or BSA-coated plates for 24 h. Then cell lysate was subjected to Western blot analysis for NICD1, PI3K-C2α, and β-actin. The right panels show the quantified data of NICD1 protein level (n = 3). Statistical significance was assessed with two-way ANOVA followed by Bonfferroni’s post-hoc test. (**c**, **f**) The effects of PI3K-C2α knockdown on Dll4-induced Notch target gene expression in HMVECs-L (**c**) and HAoSMCs (**f**). PI3K-C2α-siRNA- or ctrl-siRNA-transfected cells were incubated on Dll4- or BSA-coated plates, and 24 h later harvested for qPCR analysis (n = 3–4). Statistical significance was assessed with two-way ANOVA followed by Bonfferroni’s post-hoc test. Statistical significance was presented as *P < 0.05, **P < 0.01 or ***P < 0.001, respectively. ns: not significant.

Transfection of human umbilical vein endothelial cells (HUVECs) with PI3K-C2α-specific siRNA reduced the expression of PI3K-C2α protein by approximately 90% compared with control (ctrl)-siRNA (Fig. 1a), as reported previously^5–7^. Dll4 stimulation induced more than a sixfold increase in NICD1 level in ctrl-siRNA-transfected cells. Knockdown of PI3K-C2α reduced Dll4-induced increase in NICD1 by approximately 40% compared with ctrl-siRNA-transfected cells. A different PI3K-C2α-specific siRNA also decreased Dll4-induced NICD1 production (Supplementary Fig. S1a). Compared with Dll4, another Notch ligand Jag1 induced NICD1 production slightly, which is also inhibited by PI3K-C2α knockdown (Supplementary Fig. S1b). Quantitative PCR (qPCR) analysis showed that Dll4 increased the mRNA expression of the Notch target genes *HEY1, HEY2, HES1* and *FLT1* in control cells and that among them, PI3K-C2α knockdown inhibited Dll4-induced upregulation of *HEY1* and *HEY2* (Fig. 1b). Notch signaling regulates dynamic positive feedback loop of the expression of Notch itself^41^. HUVECs mainly expressed *NOTCH1*, *NOCTH2* and *NOCTH4* but rarely expressed *NOCTH3* (Fig. 1c and Supplementary Fig. S2a). Dll4 increased the mRNA expression of *NOTCH1* and *NOTCH3* in control cells, and PI3K-C2α knockdown inhibited Dll4-induced upregulation of *NOTCH3* but not *NOTCH1* (Fig. 1d). Because HUVECs expressed multiple Notch subtypes, we examined the involvement of Notch1 in Dll4-induced upregulation of the genes. Notch1 knockdown inhibited Dll4-induced NICD1 production (Fig. 2a) and upregulation of *HEY1*, *HEY2* and *NOTCH3* (Fig. 2b). In contrast, the forced expression of Flag-tagged NICD1 increased mRNA expression of *HEY1*, *HEY2* and *NOTCH3* compared with either non-transfected or GFP-transfected control cells (Fig. 2c and d). We also studied whether Dll4-induced Notch signaling was dependent on PI3K-C2α in another type of ECs, human microvascular ECs from lung (HMVECs-L). The expression profile of Notch paralogues in HMVECs-L was almost identical to that in HUVECs (Fig. 3a and Supplementary Fig. S2b). Knockdown of PI3K-C2α in HMVECs-L inhibited Dll4-induced NICD1 production (Fig. 3b) and the expression of its target genes *HEY1* and *HEY2* (Fig. 3c), similar to HUVECs. These results suggest that PI3K-C2α is required for Dll4-induced NICD1 production and its target gene expression via Notch1 in ECs.

We previously reported that endothelial-specific deletion of PI3K-C2α in mice resulted in embryonic lethality due to severe defects in angiogenesis whereas vascular smooth muscle-specific PI3K-C2α deletion did not impair normal growth or development^5^. Therefore, we studied the dependence of Notch signaling on PI3K-C2α in vascular SMCs. Human aortic smooth muscle cells (HAoSMCs) expressed *NOCTH1*, *NOCTH2* and *NOCTH3* but rarely expressed *NOCTH4* differently from ECs (Fig. 3d and Supplementary Fig. S2c). PI3K-C2α knockdown did not inhibit Dll4-induced NICD1 generation (Fig. 3e) or the expression of Notch target genes *HEY1* and *HES1* (Fig. 3f) in HAoSMCs. Thus, Dll4-Notch1 signaling in HAoSMCs was not dependent on PI3K-C2α, differently from the case of HUVECs.

### PI3K-C2α is not required for Dll4-induced Notch1 internalization but for cleavage of internalized Notch1

**Figure 4:**
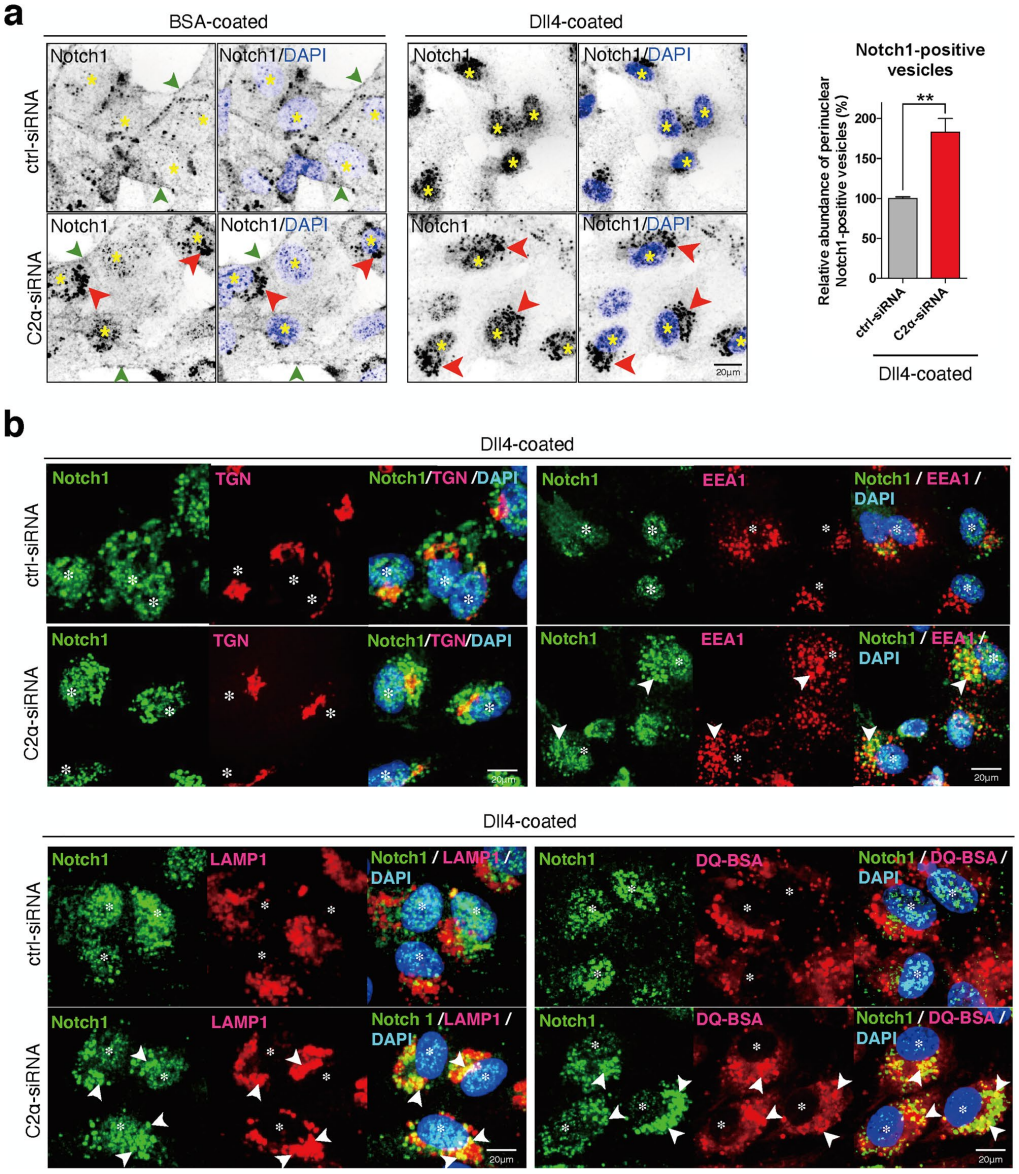
PI3K-C2α knockdown causes the perinuclear accumulation of Notch1 at the endolysosomal compartment. (**a**) Immunofluorescent staining of HUVECs with anti-Notch1 antibody (black) and nuclear DAPI staining (blue). PI3K-C2α-siRNA- or ctrl-siRNA-transfected HUVECs were incubated on Dll4- or BSA-coated dishes for 24 h. Cells were fixed and stained with anti-Notch1 antibody. Left panels, representative confocal images of anti-Notch1-stained cells and the merged views with DAPI. Right panel, quantified data of relative abundance of perinuclear Notch1-positive vesicles (n = 3). The area of perinuclear Notch1-positive vesicles was quantified with Image-J software. Statistical significance was assessed with two-tailed unpaired Student’s* t* test and presented as **P < 0.01. Notch1 immunoreactivity was detected at the plasma membrane (green arrowheads) on BSA-coated dishes and translocated to the nuclei (asterisks) upon Dll4 stimulation. Note the perinuclear Notch1-positive vesicles (red arrowheads) in PI3K-C2α-depleted cells on BSA- and Dll4-coated dishes. Scale bar, 20 μm. (**b**) Double immunofluorescent staining of Notch1 and the organelle-specific markers. PI3K-C2α-siRNA- or ctrl-siRNA-transfected HUVECs were incubated on Dll4-coated dishes for 24 h. Cells were fixed and stained with anti-Notch1 and either anti-p230 TGN (*trans*-Golgi network, top-left), anti-EEA1 (early endosome, top-right) or anti-LAMP1 (lysosome, bottom-left) antibodies. In a portion of dishes, cells were loaded with DQ Red BSA (bottom-right), which stains lysosomes, for 2 h before fixation. Nuclei were stained with DAPI. Representative confocal images illustrating the perinuclear Notch1-positive vesicles partially co-localized with EEA1- and LAMP1/DQ-BSA-positive endolysosomal compartments (white arrowheads). Asterisks denote the nuclei. Scale bar, 20 μm.

Previous studies^32–37^ revealed that ligand binding of Notch1 results in the generation of NICD1 through the formation of NEXT by ADAM-mediated proteolysis of Notch1 and subsequent γ-secretase complex-mediated cleavage of NEXT at the plasma membrane and/or the endolysosomal membrane. The γ-secretase complex-mediated NICD1 generation in the endolysosomes requires endocytosis of both Notch1 and γ-secretase complex. Because we and others showed that PI3K-C2α is involved in clathrin-mediated endocytosis^5–9,42^, we studied how PI3K-C2α is involved in NICD1 production. Anti-Notch1 immunostaining using an antibody that recognizes the intracellular domain of Notch1 showed that the Notch1 immunoreactivity was detected mainly at the plasma membrane (green arrowheads in Fig. 4a and Supplementary Fig. S3) and the perinuclear regions in ctrl-siRNA-transfected, non-stimulated cells. Dll4 stimulation of ctrl-siRNA-transfected cells induced disappearance of Notch1 at the plasma membrane and robust accumulation of Notch1 immunoreactivity in the nuclei, which likely reflected nuclear translocation of NICD1. PI3K-C2α knockdown increased Notch1-positive perinuclear dots in non-stimulated cells. In PI3K-C2α-depleted cells, Dll4 induced disappearance of plasma membrane Notch1 as in ctrl-siRNA-transfected cells, but a marked reduction in the nuclear accumulation of Notch1 immunoreactivity with enhanced Notch1-positive perinuclear dots (red arrowheads in Fig. 4a) compared with ctrl-siRNA-transfected cells. Double immunofluorescent staining using anti-Notch1 and anti-organelle-specific markers showed that Notch1-positive perinuclear dots were also positive for the *trans*-Golgi network (TGN) marker TGN, the early endosome marker EEA1, the lysosomal markers LAMP1 and DQ-BSA in ctrl-siRNA-transfected cells (Fig. 4b). PI3K-C2α knockdown resulted in substantial enhancement of Notch1 immunoreactivity in the endolysosomes (white arrowheads in Fig. 4b).

**Figure 5:**
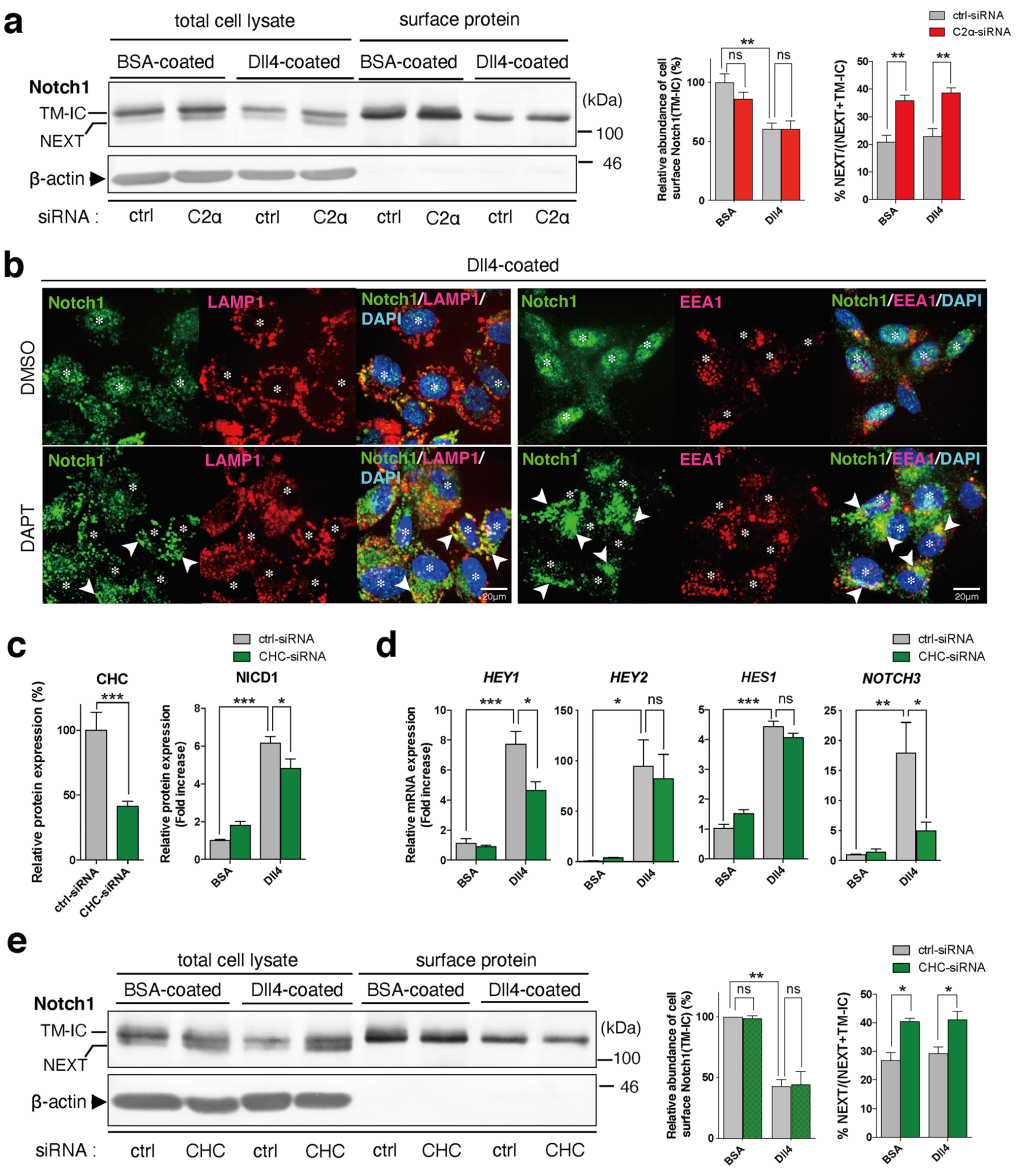
PI3K-C2α and clathrin are required for γ-secretase complex-mediated cleavage of truncated Notch1. (**a**) Representative Western blot images showing the effects of PI3K-C2α knockdown on the abundance of cell surface Notch1 and NEXT in HUVECs. PI3K-C2α-siRNA- or ctrl-siRNA-transfected cells were incubated on Dll4- or BSA-coated plates, and 24 h later total cell lysate and biotin-labeled surface proteins were prepared, followed by Western blot analysis for Notch1 and β-actin. Right panels show the quantified data of cell surface TM-IC level (n = 4) and the abundance of NEXT in total cell lysate as NEXT over NEXT + TM-IC (n = 5), respectively. Statistical significance was assessed with two-way ANOVA followed by Bonfferroni’s post-hoc test. (**b**) Representative confocal images of double immunofluorescent staining with anti-Notch1 and either anti-LAMP1 (left) or anti-EEA1 (right) antibodies. Cells received γ-secretase inhibitor DAPT (1 μM) before seeding and were incubated on Dll4-coated plates for 12 h before the fixation. Nuclei were stained with DAPI (asterisks). DAPT treatment almost completely abolished Dll4-induced nuclear translocation of Notch1. Note the endolysosomal accumulation of Notch1 (arrowheads) was observed in DAPT-treated cells. Scale bar, 20 μm. (**c**, **d**) The effects of CHC knockdown on Dll4-induced NICD1 production (**c**) and Notch target gene expression (**d**). CHC-siRNA- or ctrl-siRNA-transfected HUVECs were incubated on Dll4- or BSA-coated plates and 24 h later subjected to Western blot analysis (n = 5) and qPCR analysis (n = 3–4), respectively. Statistical significance was assessed with two-way ANOVA followed by Bonfferroni’s post-hoc test. (**e**) The effects of CHC knockdown on cell surface Notch1 level and NEXT abundance in HUVECs. CHC-siRNA- or ctrl-siRNA-transfected HUVECs were incubated on Dll4- or BSA-coated plates for 24 h. Total cell lysate and biotin-labelled surface proteins were prepared and analyzed as in (**a**). Right panels show the quantified data of cell surface TM-IC level and the abundance of NEXT in total cell lysate (n = 3). Statistical significance was assessed with two-way ANOVA followed by Bonfferroni’s post-hoc test. Statistical significance was presented as *P < 0.05, **P < 0.01 or ***P < 0.001, respectively. ns: not significant.

In cell surface-biotinylated cells, Dll4 stimulation reduced biotinylated cell surface Notch1 (TM-IC) to the similar extents in control and PI3K-C2α-depleted cells (Fig. 5a), suggesting that Dll4-induced endocytosis of Notch1 was not dependent on PI3K-C2α. It was also noted that the NEXT was more abundant in the total cell lysate of PI3K-C2α-depleted cells compared with control cells. The biotinylated surface protein fractions from either control or PI3K-C2α-depleted cells did not contain a detectable level of NEXT, which may suggest that the produced NEXT was internalized in a manner independent of PI3K-C2α. The accumulation of Notch1 immunoreactivity at the endolysosomes was mimicked by pharmacological γ-secretase inhibition using DAPT (Fig. 5b). These findings, together with PI3K-C2α dependence of Dll4-induced NICD1 production (Fig. 1a), strongly suggest that γ-secretase-mediated cleavage of NEXT but not endocytosis of NEXT is dependent on PI3K-C2α. PI3K-C2α knockdown impedes γ-secretase-mediated cleavage of internalized NEXT, resulting in the accumulation of non-cleaved NEXT in the degradation pathway endolysosomes.

Because PI3K-C2α is essential for clathrin-mediated endocytosis, we studied the effect of clathrin heavy chain (CHC) knockdown on Dll4-induced Notch signaling in HUVECs. Knockdown of CHC inhibited Dll4-induced NICD1 production (Fig. 5c). Consistently, CHC knockdown decreased Dll4-induced expression of Notch1 target genes, *HEY1* and *NOTCH3* (Fig. 5d). In cell surface-biotinylation experiments, Dll4 induced similar reductions of biotinylated cell surface Notch1 in control and CHC-depleted cells (Fig. 5e), suggesting that Dll4-induced endocytosis of Notch1 is not dependent on clathrin. In contrast, NEXT was more abundant in the total cell lysate of CHC-depleted cells (Fig. 5e), which was likely due to impaired cleavage of NEXT. Taken together, these results suggest that both PI3K-C2α and clathrin are required for Dll4-induced cleavage of internalized Notch1 but not Notch1 internalization process itself.

### PI3K-C2α is required for clathrin-dependent endocytosis of γ-secretase complex which cleaves internalized Notch1

**Figure 6:**
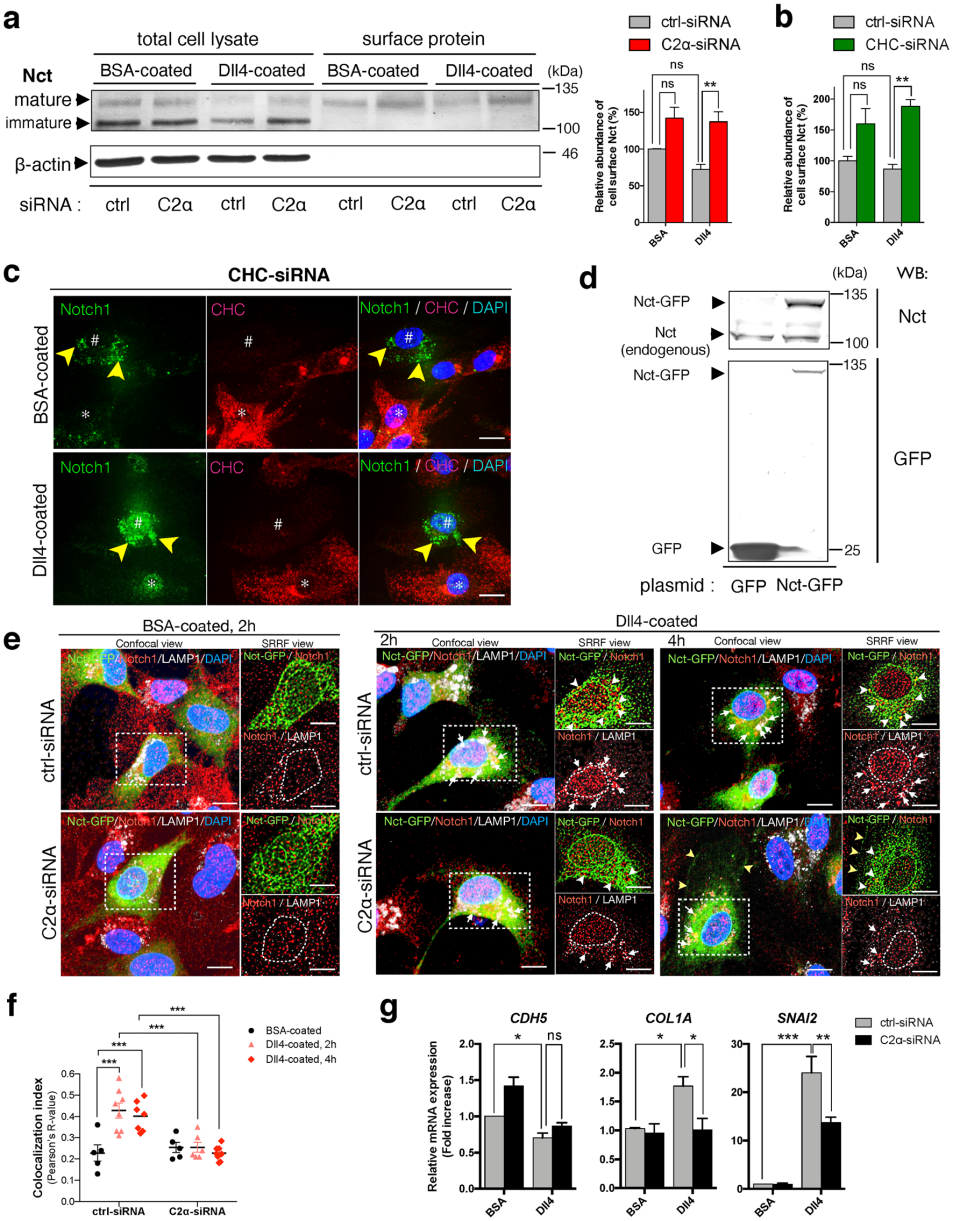
PI3K-C2α is required for clathrin-dependent endocytosis of γ-secretase complex. (**a**, **b**) The effects of PI3K-C2α (**a**) and CHC (**b**) knockdown on the cell surface Nicastrin (Nct) level in HUVECs. siRNA-transfected cells were incubated on Dll4- or BSA-coated plates for 24 h. Total cell lysate and biotin-labeled surface proteins were subjected to Western blot analysis. (n = 5). (**c**) The effects of CHC knockdown on Notch1 localization in HUVECs. CHC-siRNA-transfected cells were incubated on Dll4- or BSA-coated dishes for 24 h. Note the accumulation of Notch1 at perinuclear vesicles (yellow arrowheads) in CHC-depleted cells (#), but not in CHC-expressing cells (*). (**d**) Expression of Nct-GFP protein in HEK293T cells. Cells were transfected with Nct-GFP expression vector (2 μg) or empty vector (pAcGFP1-N, 2 μg). 12 h after transfection, cells were harvested for Western blot analysis for Nct (upper panel) and GFP (lower panel). (**e**) The effects of PI3K-C2α knockdown on the localization of Nct-GFP in HUVECs. Cells were transfected with Nct-GFP expression vector (2 μg) and either PI3K-C2α-siRNA or ctrl-siRNA, and incubated on Dll4- or BSA-coated dishes for 2 h or 4 h. The SRRF views of the boxed regions in confocal views are also shown in the right upper and lower of each panel. Note the intracellular colocalization of Nct-GFP and Notch1 (white arrowheads) at LAMP1-positive lysosomes (white arrows), which was more abundant in control cells compared to PI3K-C2α-depleted cells. Nct-GFP was also accumulated at the cell surface of PI3K-C2α-depleted cells (yellow arrowheads). Dashed lines in the SRRF views (right lower of each panel) delineate the nuclei. (**f**) Quantification of colocalization efficiency of Nct-GFP and Notch1. The colocalization index was quantified using Image-J software. Pearson’s R-value is shown (n = 5–8). (**g**) The effects of PI3K-C2α knockdown on Dll4-induced expression of EndMT-related genes in HUVECs. PI3K-C2α-siRNA- or ctrl-siRNA-transfected cells were incubated on Dll4- or BSA-coated plates and 24 h later harvested for qPCR analysis (n = 5). Statistical significance was assessed with two-way ANOVA followed by Bonfferroni’s post-hoc test in (**a**, **b**, **f**, **g**). Statistical significance was presented as *P < 0.05, **P < 0.01 or ***P < 0.001, respectively. ns: not significant.

We tested the hypothesis that PI3K-C2α might be necessary for endocytosis of γ-secretase complex that cleaves internalized Notch1 at the endolysosomal compartment. We explored whether PI3K-C2α was required for endocytosis of nicastrin (Nct), a core component of γ-secretase complex^43^. Knockdown of PI3K-C2α increased the cell surface glycosylated mature form of Nct to the similar extents in non-stimulated and Dll4-stimulated cells (Fig. 6a). Likewise, knockdown of CHC increased the amount of the cell surface Nct in non-stimulated and Dll4-stimulated cells (Fig. 6b). These findings suggested that Nct-containing γ-secretase complex is endocytosed in both non-stimulated and Dll4-stimulated conditions in PI3K-C2α- and clathrin-dependent manners. We studied the effects of CHC knockdown on nuclear and perinuclear accumulation of Notch1 immunoreactivity. Perinuclear accumulation of Notch1 immunoreactivity was observed only in CHC-depleted cells but not in non-depleted cells in the same microscopic field (Fig. 6c). We next examined the effects of PI3K-C2α knockdown on endocytosis of GFP-tagged Nct (Nct-GFP) and colocalization of Nct-GFP and Notch1 immunoreactivity and on the nuclear accumulation of Notch1 immunoreactivity (Fig. 6d and e). We took advantage of exogenous Nct-GFP expression because commercially available anti-Nct antibodies were not properly validated for immunostaining of Nct. With high resolution confocal microscope, in non-stimulated, control siRNA-transfected cells on the BSA-coated dish, expressed Nct-GFP was observed in a punctate pattern diffusely in the cytoplasm and in a mesh-like pattern at the perinuclear region, and was hardly colocalized with Notch1 immunoreactivity, which was distributed in a punctate pattern in the cytoplasm and moderately in the nucleus (Fig. 6e). In contrast, in Dll4-stimulated, ctrl-siRNA-transfected cells, the colocalization of Nct-GFP and Notch1 immunoreactivity was observed at the perinuclear region, most of which were LAMP1-positive lysosomes (white arrowheads in Fig. 6e), at 2 h and 4 h after seeding. Dll4 stimulation also increased nuclear Notch1 immunoreactivity, which most likely represented NICD1. PI3K-C2α knockdown inhibited the perinuclear colocalization of Nct-GFP and Notch1 immunoreactivity and the nuclear accumulation of Notch1 immunoreactivity (SRRF views of Fig. 6e and f). In addition, PI3K-C2α knockdown resulted in the accumulation of Nct-GFP at the cell surface (yellow arrowheads at 4 h on the Dll4-coated dish in Fig. 6e) probably because of inhibited endocytosis of Nct-GFP.

### PI3K-C2α mediates Dll4-induced expression of mesenchymal cell marker in HUVECs

Dll4 stimulation increased the expression of Notch3 in HUVECs, which was inhibited by PI3K-C2α depletion (Fig. 1d). Notch3 is predominantly expressed in SMCs but is rarely expressed in ECs in a non-stimulated condition^44^. It is reported that upregulation of Notch3 in ECs results in endothelial to mesenchymeal transition (EndMT)^45^. We investigated the expression of EndMT-related genes in HUVECs. Dll4 stimulation decreased the expression of the endothelial cell marker, *CDH5* (VE-cadherin) and increased the expression of the mesenchymal cell marker *COL1A* in HUVECs. PI3K-C2α knockdown reversed these Dll4-induced changes, although the difference of only *COL1A* expression reached the statistical significance (Fig. 6g). It is reported that transcriptional repressor *SNAI2* (also known as *SLUG*) mediates Notch-induced EndMT^46^. Dll4 stimulation increased *SNAI2* expression in HUVECs, which was significantly impaired by PI3K-C2α depletion (Fig. 6g). These observations raised the possibility that PI3K-C2α is functionally involved in EndMT induced by Dll4-Notch signaling.

## Discussion

**Figure 7:**
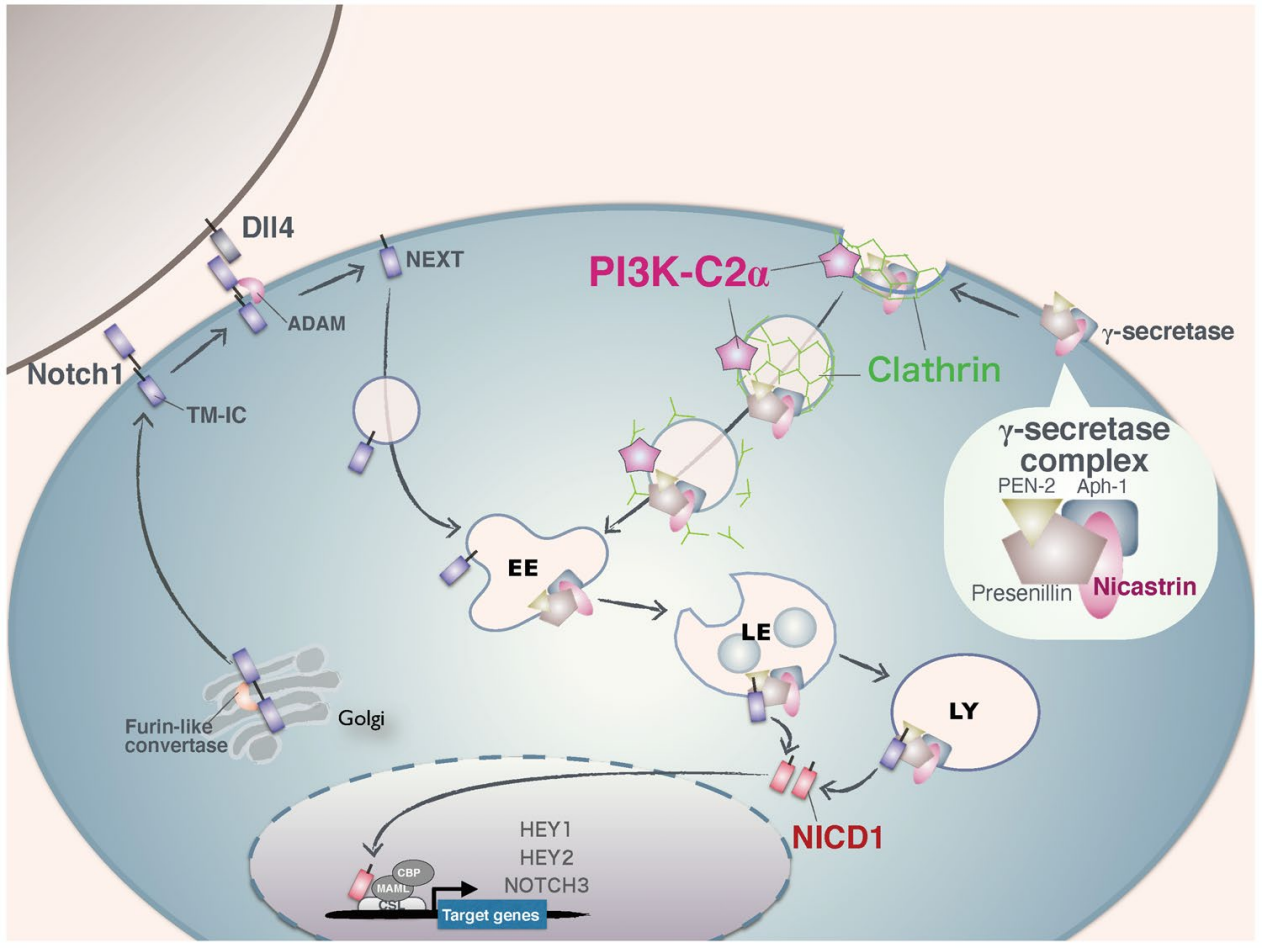
Schematic summary showing the role of PI3K-C2α in Dll4-Notch1 signaling in ECs. After the processing by Furin-like convertases (S1 cleavage) in the Golgi, cleaved Notch receptors, which are now heterodimers composed of the extracellular domain and TM-IC, are delivered to the plasma membrane. γ-secretase complexes, which are hetero-tetramers composed of Presenilin, Nct, PEN-2 and Aph-1, exist at the plasma membrane and are constitutively internalized in a manner dependent on clathrin and PI3K-C2α. Dll4 binding induces Notch1 cleavage at the juxtamembrane site (S2 cleavage) by ADAM to generate NEXT. NEXT is internalized in a manner independent of clathrin and PI3K-C2α. Internalized NEXT is cleaved at the transmembrane domain (S3 cleavage) by γ-secretase at the endolysosomal compartment to release NICD1. NICD1 translocates to the nucleus and forms the transcriptional activator complex to stimulate the expression of Notch target genes including *HEY1*, *HEY2* and *NOTCH3*. EE: early endosomes, LE: late endosomes, LY: lysosomes.

In this study, we found that PI3K-C2α was required for Dll4-induced NICD1 production and the expression of Notch-target genes in HUVECs. Ligand-induced NICD1 generation in HUVECs involved endocytosis of ligand-activated Notch1 and γ-secretase complex. PI3K-C2α was necessary for the endocytosis of γ-secretase complex but not Notch1. The internalized γ-secretase complex mediated proteolytic cleavage of endocytosed Notch1 to generate NICD1. Thus, our study suggests that PI3K-C2α is required for intracellular activation of Notch1 by mediating endocytosis of γ-secretase complex in ECs (Fig. 7).

PI3K-C2α knockdown decreased Dll4-induced production of NICD1 and the expression of Notch1 target genes in human vascular EC types, HUVECs and HMVECs-L (Figs. 1a, b, 3b and c). The present study, together with our previous reports^5–7^, showed that PI3K-C2α is required for endocytosis-mediated signaling of several different classes of cell surface receptors. In our previous studies, we showed that PI3K-C2α is required for endosomal receptor signaling, which includes Rac activation by the G-protein-coupled receptor S1P_1_, Rho activation by the receptor tyrosine kinase VEGFR2 and Smad phosphorylation by the TGFβ1 receptor serine/threonine kinase ALK5, through being involved in endocytosis of these cell surface receptors. Differently from the cases of these cell surface receptors, PI3K-C2α in Notch signaling is required for endocytosis of the Notch-cleaving protease γ-secretase complex but not Notch receptor itself. By this action, PI3K-C2α is necessary for the generation of NICD1, an essential mediator of Notch signaling in ECs. In the present study, PI3K-C2α knockdown or pharmacological γ-secretase inhibition resulted in an increase of Notch1 immunoreactivity at the perinuclear endolysosomes, which implied the accumulation of internalized Notch1 that escaped proteolysis by γ-secretase complex (Figs. 4 and 5b).

After ligand binding, Notch receptors are finally cleaved by γ-secretase complex at the cell surface^40^ or at the intracellular vesicles to release NICD1^32,33^. γ-secretase complex is a member of the intramembrane cleaving proteases, which proteolyse type I transmembrane proteins at the hydrophobic compartment of the transmembrane domain. γ-secretase is hetero-tetrameric complex composed of presenilin (PS), Nct, anterior pharynx defective 1 (APH-1) and presenilin enhancer 2 (PEN-2). PS possesses a catalytic domain and has two paralogues, PS1 and PS2. There are also two APH-1 genes, *APH1A* and *APH1B*, in humans. Thus, at least four different γ-secretase complexes exist in humans^47,48^. All four components are required for γ-secretase activity and active γ-secretase complexes contain each component in a 1:1:1:1 stoichiometry^49–52^. Nct functions as a substrate receptor in the γ-secretase complex^53^. Because there is no isoform of Nct, it is present in all γ-secretase complexes. In the present study, knockdown of either PI3K-C2α or CHC inhibited internalization of Nct with or without Dll4 stimulation (Fig. 6a, b, e and f), suggesting that PI3K-C2α is required for endocytosis of γ-secretase complex, which occurs in a manner independent of Dll4 stimulation. In contrast, knockdown of PI3K-C2α or CHC did not alter endocytosis of the cell surface Notch1 but rather increased the cellular protein level of ADAM-truncated Notch1, NEXT (Fig. 5a and e). Moreover, knockdown of PI3K-C2α or CHC induced the accumulation of Notch1 immunoreactivity at the endolysosomes, as well as the γ-secretase inhibitor DAPT (Figs. 4, 5b and 6c). Taken together, PI3K-C2α is required for clathrin-dependent endocytosis of γ-secretase complex but not Notch1, and the internalized γ-secretase complex mediates the final step of Notch1 cleavage to generate NICD1 likely at the endolysosomal compartments. When endocytosis of γ-secretase complex is impaired by knockdown of PI3K-C2α and CHC or γ-secretase activity is inhibited pharmacologically, γ-secretase-mediated cleavage of internalized Notch1, most likely NEXT, is hampered with the accumulation of Notch1 in the endolysosomes. In the present study, we could not determine the molecular entitiy of Notch1 immunoreactivity at the endolysosomes because cleavage-specific anti-Notch1 antibodies were not available. Further studies are required to exactly identify the molecular entity of Notch1 at the endolysosomes.

After translation, γ-secretase components undergo several maturation steps such as complex assembly, glycosylation of Nct and endoproteolysis of PS. Then, mature γ-secretase complexes are delivered to the cell surface or endosomes^47^. Indeed, growing evidence suggests that fully assembled γ-secretase complexes are localized at the plasma membrane and lysosomes^54–56^. Previous study reported that the mature form of γ-secretase is constitutively endocytosed from cell surface via clathrin-mediated pathway in a manner dependent on Clathrin Assembly Lymphoid Myeloid leukemia (CALM)^38^. PI3K-C2α is recruited to clathrin-coated pits through its N-terminal clathrin-binding domain to facilitate growth of clathrin-coated pits^9^. It is known that the proteolytic activity of γ-secretase is higher at low pH conditions^55^ and impairment of lysosomal acidification disrupts the γ-secretase-mediated cleavage of Notch at endolysosomal compartments^57,58^. Therefore, PI3K-C2α-mediated delivery of γ-secretase complex to the endolysosomes provides a suitable condition of efficient Notch1 proteolysis by γ-secretase complex for generating NICD1. Because PI3K-C2α knockdown does not impair endocytosis of Notch1, the suppression of γ-secretase endocytosis results in the retention of Notch1 immunoreactivity at the endolysosomes, as demonstrated in Fig. 4. Although PI3K-C2α depletion caused the accumulation of Notch1 at endolysosomal compartments, the condition of PI3K-C2α deficiency did not result in the activation of Notch signaling probably because γ-secretase was absent at that compartment.

Some previous papers reported Notch1 is internalized through clathrin-dependent manner^32,36^. In contrast, other reports suggest that endocytosis in not required for Notch signaling^39,40^. It is also reported that there are several endocytic routes in Notch1 internalization^33^. These reports raise the possibility that Notch1 internalization pathway is cell-type- or context-dependent. It is also possible that several Notch1 internalization pathways coexist and orchestrate the signal intensity. PI3K-C2α knockdown inhibits γ-secretase-mediated Notch1 cleavage at the endolysosomes, but may not impair Notch1 cleavage at the cell surface. Therefore, PI3K-C2α knockdown-induced reductions of NICD1 production and target gene expression may be partial (Fig. 1a and b). Further studies are required to fully understand the mechanism of PI3K-C2α-dependent NICD1 generation.

The involvement of the PI3K-C2α in Notch signaling is cell type-specific: Dll4-induced NICD1 production was not dependent on PI3K-C2α in vascular SMCs, differently from ECs (Fig. 3). Although the mechanisms underlying the cell type-specific differences are not fully understood, it might be due to the possible cell type-specific compensation of PI3K-C2α deficiency by another class II PI3K member PI3K-C2β. Alternatively, it might be due to the difference of the cell site, i.e. the endolysosomes or the plasma membrane, where γ-secretase complex proteolyses Notch1 to generate NICD1, in vascular SMCs and ECs. If the plasma membrane could be the major site of γ-secretase complex-mediated cleavage of Notch1 to generate NICD1, Notch1 cleavage would not require endocytosis of γ-secretase complex and, therefore, PI3K-C2α.

Notch ligands Dlls and Jagged are transmembrane molecules, as well as Notch. In the in vivo conditions, membrane trafficking may regulate not only Notch receptors in target cells but also Notch ligands in the neighboring cells. In the present study, we immobilized Dll4 onto plates or dishes as a means to stimulate Notch on seeded HUVECs. This allowed us to focus on membrane trafficking of Notch receptors in Dll4 signal-receiving cells. It would be intriguing to study a possible involvement of PI3K-C2α in the regulation of the endocytosis of Notch ligands.

PI3K-C2α deficiency causes the defects in sprouting angiogenesis and vascular maturation resulting in the embryonic lethality^5^. We have previously reported that loss of PI3K-C2α disrupts several signaling pathways including VEGF, S1P and TGFβ, which are the possible causes of PI3K-C2α deletion phenotypes^5–7^. Notch1-null mouse embryo is also reported to show the vascular maturation defect^20^. This phenotype is similar to PI3K-C2α-null embryo, raising the possibility that PI3K-C2α-mediated Notch signaling is involved in the developmental vessel maturation. Finally, we investigated the possible involvement of PI3K-C2α in Dll4-mediated EndMT because Notch3 expression in ECs, which is caused by Dll4 stimulation (Fig. 1d), is reported to be involved in EndMT progression^45^. We revealed that PI3K-C2α was required for Dll4-induced expression of EndMT regulator *SNAI2* as well as the downregulation of endothelial cell marker *CDH5* and the upregulation of mesenchymal cell marker *COL1A* induced by Dll4 stimulation (Fig. 6g). It is reported that Dll4-Notch mediated EndMT is required for cardiac cushion development^46^. Further in vivo studies are needed to investigate the function of PI3K-C2α as a regulator of Notch signaling.

In summary, the present study indicates that PI3K-C2α is essential for clathrin-mediated endocytosis of γ-secretase complex and Dll4-induced cleavage of Notch1 at the endolysosomal compartment, which is followed by NICD1 production and target gene expression in ECs. Thus, PI3K-C2α is a novel mediator of Notch signaling pathway by functioning as an endocytosis regulator of γ-secretase complex. Further studies to elucidate the significance of PI3K-C2α in Notch signaling pathway provide new insights into previously unexplained diversification of organ development.

## Methods

### Cell culture and small interfering RNA (siRNA) transfection

HUVECs (Lonza, Basel, Switzerland), HMVECs-L (Lonza), HAoSMCs (Lonza) and human embryonic kidney (HEK) 293 T cells were cultured on type I collagen (Nitta Gelatin, Osaka, Japan)-coated flasks and dishes and grown under 5% CO2 in the humidified air at 37℃ in the appropriate media, respectively. HUVECs were cultured in EGM-2 supplemented with 2% FBS and growth factor supplement cocktail (#CC3156, Lonza). HMVECs-L were cultured in EGM-2 MV (#CC4147, Lonza) supplemented with 2% FBS and growth factor supplement cocktail. HAoSMCs were cultured in SmGM-2 (#CC3182, Lonza) supplemented with 5% FBS and growth factor supplement cocktail. HEK 293 T cells were cultured in DMEM (#044–29765, Wako, Osaka, Japan) supplemented with 10% FBS (Gibco, Waltham, MA). Cells of passage 5 or 6 were used for all experiments. siRNAs used in the experiments were synthesized using a Silencer siRNA construction kit (#AM1620; Ambion, Austin, TX) according to the manufacture’s protocol. The human PI3K-C2α#1 target sequence was 5′-AAGGTTGGCACTTACAAGAAT–3′. The human PI3K-C2α#2 target sequence was 5′-AAGTAAGCCTAAGGTGGATAA–3′. The human Notch1 target sequence was 5′-AAGGTGTCTTCCAGATCCTGA-3′. The human CHC target sequence was 5′-AATCCAATTCGAAGACCAATT-3′. As a control, scrambled-siRNA^5^ or negative-control-siRNA (#1022076, Qiagen, Hilden, Germany) were used. PI3K-C2α-siRNA#1 was used as PI3K-C2α-siRNA unless stated otherwise. Cells were cultured to 70% confluency on the culture dishes and then medium was changed to OptiMEM (Gibco). Subsequently, the cells were transfected with 20 nM of siRNA using Lipofectamine RNAiMAX (Invitrogen, Waltham, MA) according to the manufacturer’s protocol. 4 h after transfection, medium was replaced with the complete growth medium. The cells were cultured for further 24–48 h before processing for each experiment.

### Notch pathway activation

Before seeding cells, cell culture dishes, plates and 8-well Lab-Tek Chamber Slides (Thermo Scientific, Waltham, MA, USA) were coated with recombinant human DLL4 (R&D systems, Minneapolis, MN, USA, 2 μg/mL), recombinant human Jag1 (R&D systems, 2 μg/mL) or bovine serum albumin (BSA) dissolved in sterile Ca^2+^- and Mg^2+^-free phosphate-buffered saline (PBS) for 1 h at room temperature. Then, the solutions were removed and cells were seeded onto coated dishes, plates and slides. Cells were seeded and incubated in complete growth medium under 5% CO_2_ at 37℃ for the indicated time periods. In Fig. 5b, 1 μM of γ-secretase inhibitor DAPT (#565784, Millipore, Billerica, MA, USA, 1 μM) was added to suspended cells before cell seeding. To overexpress NICD1, HUVECs were transfected with 3xFlag-tagged murine NICD1 expression plasmids (Addgene plasmid #20183, 2–4 μg) using Amaxa HUVEC Nucleofector Kit. Transfected cells were subjected to the immunoblot or quantitative PCR analysis, 24 h later.

### Western blotting

After the incubation on the Dll4- or BSA-coated plates, cells were washed with PBS and lysed with 2 X Laemmli’s solubilizing buffer [100 mM Tris (pH 6.8), 2% SDS, 0.008% Bromophenol Blue, 2% 2-mercaptoethanol, 26.3% glycerol and 0.001% Pyronin Y]. Cell lysate was subjected to 8% SDS-PAGE and separated proteins were transferred onto PVDF membranes (Millipore). The membranes were blocked with PVDF Blocking reagent (Toyobo, Osaka, Japan) and then some membranes were devided into two or three pieces. Each part of the cut membrane pieces or uncut whole membrane was incubated with respective primary antibodies at 4℃ overnight. Primary antibodies used were as follows: PI3K-C2α (#12402, Cell Signaling Technology, Danvers, MA, USA, 1:1000), Notch1 (#3608, Cell Signaling Technology, 1:1000), Cleaved-Notch1 (NICD1) (#4147, Cell Signaling Technology, 1:1000), DDDDK(Flag)-tag (#M185-3L, Medical & Biological Laboratories Co., Ltd., Nagoya, Japan, 1:1000), CHC (#MA1-065, Thermo Scientific, 1:1000), Nct (#9447, Cell Signaling Technology, 1:1000), GFP (#sc-9996, Santa Cruz Biotechnology, Inc., CA, USA, 1:1000), β-actin (#013–24553, Wako, 1:1000). The membranes were incubated with alkaline phosphatase-conjugated secondary antibodies (anti-mouse IgG antibody, #7056, Cell Signaling Technology, 1:1000. anti-rabbit IgG antibody, #7054; Cell Signaling Technology, 1:1000) and visualized by color reaction using 5-bromo-4-chloro-3-indolyl-phosphate/nitro blue tetrazolium (Wako). In Fig. 5, electrophoresis and color reaction was conducted for longer time to clearly identify TM-IC and NEXT bands. The band intensities were analysed using Image Studio Lite (v5.0.21, LI-COR Bioscience, Lincoln, NE, USA, https://www.licor.com/bio/image-studio-lite/). The values were normalized for the value of β-actin as a loading control and expressed as multiples over the normalized values of untreated controls. See Supplementary Fig. S4, which shows the original membranes before the cropping and the cropped regions.

### RNA isolation and quantitative PCR (qPCR) analysis

Total RNA was isolated from cultured cells using TRIzol reagent (Invitrogen). 0.5–1.0 μg of total RNA was reverse-transcribed into cDNA using QuantiTect RT Kit (#205311, Qiagen). Quantitative real time PCR analyses were performed using FastStart Universal SYBR Green Master (#04913914001, Roche, Basel, Switzerland) and GeneAmp 7300 system (Applied Biosystems, Foster City, CA, USA) as previously described^59^. Threshold value was determined automatically within the exponential growth region and threshold cycle (Ct) value was defined as cycle number at which fluorescence passed the threshold. ΔCt value was determined by subtracting the Ct value of *ACTB* (β-actin gene) from that of the target gene. ΔΔCt value was calculated by subtracting the ΔCt value of ctrl-siRNA-transfected, non-stimulated control cells from the respective ΔCt value of each experimental condition. 2^-ΔΔCt^ was presented as a fold increase against control cells in the figures. Semi-quantification of *NOTCH1-4* expression levels was performed by subtracting the ΔCt value of *NOTCH1* from that of *NOTCH1-4* to calculate the ΔΔCt value and (2^-ΔΔCt^ × 100) (%) was presented as the relative abundance of *NOTCH1-4* mRNA expression against *NOTCH1* in Figs. 1c, 3a and d and Supplementary Fig. S2. Determination of PCR amplification efficiency of *NOTCH1-4* cDNAs was performed using serially diluted cDNA samples from Dll4-stimulated HUVECs. Regression lines and amplification efficiency were determined based on the Ct values of each diluted sample using Prism 6 software (v6.0 h, GraphPad Software, Inc., https://www.graphpad.com/scientific-software/prism/). Amplification efficiency (%) was determined by the formula of ((10^–1/slope^ – 1) × 100) using the slope of a regression line. Because amplification efficiency was very similar between *NOTCH1-4* (Supplementary Fig. S2d), we did not incorporate it into the assessment of the relative mRNA abundance. Primers used in the experiments are listed in Supplementary Table S1.

### Immunofluorescent staining and lysosome labeling with DQ-BSA

To detect the proteolytic activity of lysosomes, cells were incubated with DQ Red BSA (#D12051, Life Technologies, Waltham, MA, USA, 10 μg/mL) for 2 h before fixation in Fig. 4b. For immunofluorescent staining, cells were washed with pre-warmed Ca^2+^- and Mg^2+^-containing PBS (PBS ( +)) and fixed with pre-warmed 4% paraformaldehyde for 15 min. After fixation, cells were washed with PBS and incubated with 5% normal goat serum and 0.3% Triton X-100 containing PBS for 1 h at room temperature. Then, cells were incubated with primary antibodies at 4℃ overnight. Primary antibodies used were as follows: human Notch1 (#3608, Cell Signaling Technology, 1:400), human Na^+^/K^+^ ATPase α1 (#sc-21712, Santa Cruz Biotechnology, 1:400), human EEA1 (#610456, BD Biosciences, 1:500), human p230 trans-Golgi (#611280, BD Biosciences, 1:500), human LAMP-1 (#328601, BioLegend, San Diego, CA, USA, 1:500), human CHC (#MA1-065, Thermo Scientific, 1:1000). Cells were washed with 0.1% Triton X-100 containing PBS and incubated with secondary antibodies for 1.5 h at room temperature. The secondary antibodies used are Alexa Fluor 488-conjugated goat anti-rabbit IgG (#A11034, Life Technologies, Waltham, MA, USA, 1:1000) and Alexa Fluor 594-conjugated goat anti-mouse IgG (Molecular Probes, Waltham, MA, USA, 1:1000). Cells were counterstained with DAPI (#D1306, Molecular Probes) for 15 min. Cells were mounted using Fluoromount (#K024, Diagnostic BioSystems, Pleasanton, CA, USA) and observed under a custom confocal microscope unit as described in detail previously^5,61^.

### Biotinylation of cell surface protein

Cells on the Dll4- or BSA-coated dishes were washed with ice-cold PBS twice and then treated with 1.0 mg/mL of EZ-link sulfo-NHS-SS-biotin (#21331, Thermo Scientific) for 15 min on ice. Biotinylation reactions were terminated with 50 mM glycine in PBS. After washing with PBS, cell extracts were prepared in radioimmunoprecipitation assay (RIPA) buffer [10 mM Tris–HCl (pH7.4), 100 mM NaCl, 1 mM EDTA, 1 mM EGTA, 1 mM NaF, 20 mM Na_4_P_2_O_4_, 2 mM Na_3_VO_4_, 0.1% SDS, 0.5% sodium deoxycholate, 1% Triton-X 100, 10% glycerol] with the protease inhibitor cocktail cOmplete, Mini (#11836153001, Roche). Cell extracts were sonicated and centrifuged at 8,000 × g at 4℃ for 30 min. Aliquots were made from supernatant and used as “total cell lysate” in the immunoblotting analysis. The rest of the supernatant was mixed with PierceTM NutrAvidinTM Agarose (#29,200, Thermo Scientific) and rotated for 2–3 h at 4 ℃. The beads were then washed with RIPA buffer three times and biotinylated proteins were eluted with 2 × Laemmli’s buffer at 65℃ for 10 min. Total cell lysate and biotinylated surface proteins were subjected to immunoblot analysis.

### Construction of Nct-GFP expression plasmid and super-resolution radial fluctuation-Stream imaging

Human nicastrin (Nct) cDNA fragments were amplified by PCR using human NCSTN cDNA clone plasmid (Sino Biological Inc., Beijing, China) as a template and PrimeSTAR HS DNA Polymerase (#R010A, Takara Bio Company, Shiga, Japan). Sense and antisense primers used was 5′-AAGGCCTCTGTCGACATGGCTACGGCAGGGGGT-3′and 5′-AGAATTCGCAAGCTTGTATGACACAGCTCCTGGCTC-3′, respectively. The expression vector of Nct-GFP, in which Nct was tagged with enhanced green fluorescent protein at the C-terminus, was generated by ligating the PCR products into pAcGFP1-N vector (#632501, Clontech Laboratories Inc, Shiga, Japan), using In-Fusion HD Cloning Kit (#Z9633N, Takara). The accuracy of Nct-GFP sequence was confirmed by restriction enzyme digestion and nucleotide sequencing.

HUVECs were transfected with Nct-GFP expression vector (2 μg) and either PI3K-C2α-siRNA or ctrl-siRNA, using Amaxa HUVEC Nucleofector Kit (#VPB-1002, Lonza). 2 h or 4 h after seeding, cells were washed with pre-warmed PBS ( +) and fixed with pre-warmed 4% paraformaldehyde for 15 min. Cells were incubated with primary antibodies, anti-human Notch1 (#3608, Cell Signaling Technology, 1:400) and anti-human LAMP-1 (#328601, BioLegend, 1:500) at 4℃ overnight. Cells were washed with 0.1% Triton X-100 containing PBS and incubated with secondary antibodies, Alexa Fluor 568-conjugated goat anti-rabbit IgG (#A11011, Invitrogen, 1:1000) and Alexa Fluor 647-conjugated goat anti-mouse IgG1 (#A21240, Invitrogen, 1:1000) for 1.5 h at room temperature. Finally, cells were counterstained with DAPI (#D1306, Molecular Probes) for 15 min. Then cells were analysed using a super-resolution microscopy as described previously^42,60^. Briefly, Super-resolution imaging was performed using a Dragonfly confocal microscope in super-resolution radial fluctuation (SRRF)-Stream mode (Andor Technology Ltd.-Oxford, UK). The colocalization index was quantified using Image-J software (v1.48, https://imagej.nih.gov/ij/download.html).

### Statistical analysis

Statistical analysis was performed with Prism 6 software. Data are presented as mean ± standard error of mean (SEM). Paired data were evaluated with two-tailed unpaired Student’s* t* test and comparison of multiple groups was performed using two-way ANOVA followed by Bonfferroni’s post-hoc test, unless stated otherwise. *P* < 0.05 was considered to be statistically significant. Statistical significance was presented as * (*P* < 0.05), ** (*P* < 0.01) or *** (*P* < 0.001) respectively.

## Supplementary information

Supplementary material 1

## Data Availability

All data described in the manuscript are contained within the manuscript and available from the corresponding author on reasonable request.
