# Supplementary material for: Class II phosphatidylinositol 3-kinase-C2α is essential for Notch signaling by regulating the endocytosis of γ-secretase in endothelial cells
Source: Sci Rep. 2021 Mar 4;11:5199. doi: 10.1038/s41598-021-84548-4 (PMC7933152; doi:10.1038/s41598-021-84548-4)
Supplement: Supplementary file 1 — Supplementary material 1 [file 41598_2021_84548_MOESM1_ESM.pdf]

Supplementary Information

Class II phosphatidylinositol 3-kinase-C2α is essential for Notch signaling by regulating the endocytosis of γ-secretase in endothelial cells

Shota Shimizu, Kazuaki Yoshioka, Sho Aki and Yoh Takuwa

Supplementary Table S1. Primers used in this study

Gene	Forward (5'>3')	Reverse (5'>3')
Hey1	CATACGGCAGGAGGGAAAG	GCATCTAGTCCTTCAATGATGCT
Hey2	CCCGCCCTTGTCAGTATC	TTGTTTGTTCCACTGCTGGT
Hes1	GAAGCACCTCCGGAACCT	GTCACCTCGTTCATGCACTC
Flt1	ATGCCAGCAAGTGGGAGTT	CAAAAGCCCCTCTTCCAAGT
Flk1	GAACATTTGGGAAATCTCTTGC	CGGAAGAACAATGTAGTCTTTGC
Notch1	CGGGGCTAACAAAGATATGC	CACCTTGGCGGTCTCGTA
Notch2	CAACCGCAATGGAGGCTATG	GCGAAGGCACAATCATCAATGTT
Notch3	TGGCGACCTCACTTACGACT	CACTGGCAGTTATAGGTGTTGAC
Notch4	TGTGAACGTGATGTCAACGAG	ACAGTCTGGGCCTATGAAACC
Cdh5	AGGACGCTTTCACCATTGAG	TCATGTATCGGAGGTCGATG
Col1a	GAACGCGTGTCATCCCTTGT	GAACGAGGTAGTCTTTCAGCAACA
Snai2	CGAACTGGACACACATACAGTG	CTGAGGATCTCTGGTTGTGGT
β-actin	TCTACAATGAGCTGCGTGTG	ATGGCTGGGGTGTTGAAG

Supplementary Figure S1.

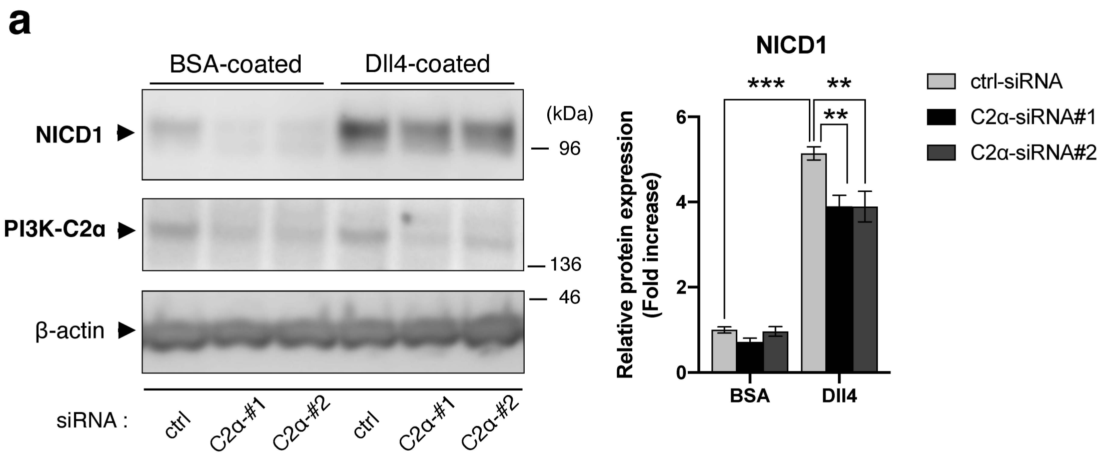

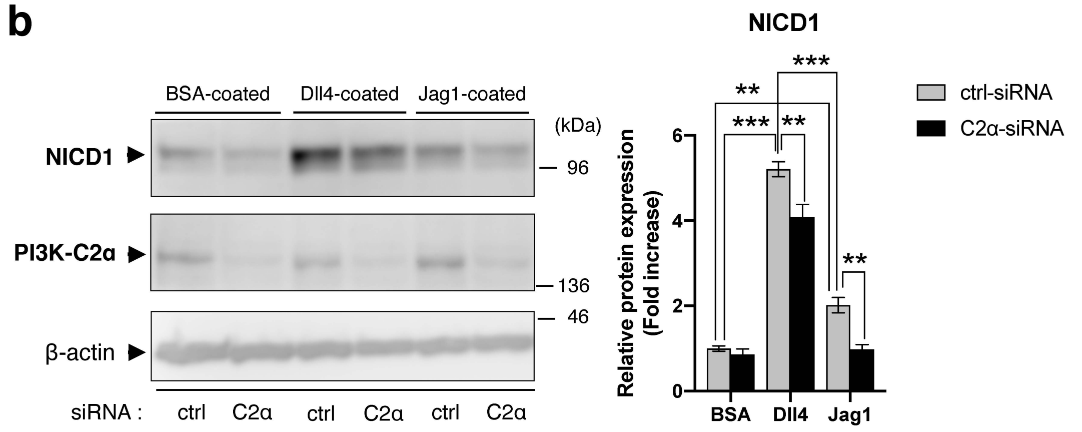

Supplementary Figure S1. PI3K-C2α is required for Notch signaling in HUVECs.
(**a**) The effects of two different PI3K-C2α-specific siRNAs on Dll4-induced NICD1 production. HUVECs were transfected with PI3K-C2α-specific siRNAs #1 or #2 or ctrl-siRNA, stimulated with Dll4 or not, and 24 h later underwent Western blot analyses using anti-NICD1, anti- PI3K-C2α and anti-β-actin antibodies. (**b**) The effects of PI3K-C2α knockdown on Dll4- and Jag1-induced NICD1 production in HUVECs. HUVECs were transfected with PI3K-C2α-specific siRNA #1 or ctrl-siRNA, stimulated with Dll4 or Jag1 or without ligand stimulation, and 24 h later underwent Western blot analyses as in (a). In (a) and (b), representative Western blot images are shown on the left and the quantified data are on the right (n=4). $^{**}P < 0.01$ and $^{***}P < 0.001$ as assessed with two-way ANOVA followed by Bonfferroni's post-hoc test.

Supplementary Figure S2.

a

HUVECs

Target gene	Ct	ΔCt	ΔΔCt	($2^{-\Delta\Delta Ct}$) x100
NOCTH1	22.7 ± 0.2	4.5 ± 0.1	0	100%
NOCTH2	24.5 ± 0.2	6.4 ± 0.1	1.8 ± 0.1	28.3 ± 2.4
NOCTH3	32.7 ± 0.3	14.6 ± 0.3	10.0 ± 0.3	0.1 ± 0.0
NOCTH4	24.9 ± 0.2	6.8 ± 0.1	2.3 ± 0.1	21.0 ± 1.1
ACTB	18.1 ± 0.1			

b

HMVECs-L

Target gene	Ct	ΔCt	ΔΔCt	($2^{-\Delta\Delta Ct}$) x100
NOCTH1	23.9 ± 0.3	7.0 ± 0.2	0	100%
NOCTH2	25.4 ± 0.4	8.5 ± 0.4	1.5 ± 0.3	37.9 ± 8.4
NOCTH3	31.2 ± 0.7	14.3 ± 0.6	7.3 ± 0.4	0.7 ± 0.2
NOCTH4	24.3 ± 0.4	7.4 ± 0.3	0.4 ± 0.2	78.2 ± 9.1
ACTB	16.9 ± 0.1			

c

HAoSMCs

Target gene	Ct	ΔCt	ΔΔCt	($2^{-\Delta\Delta Ct}$) x100
NOCTH1	26.1 ± 0.4	9.0 ± 0.2	0	100%
NOCTH2	23.0 ± 0.2	6.0 ± 0.1	-3.1 ± 0.2	846.0 ± 120.2
NOCTH3	23.7 ± 0.4	6.7 ± 0.1	-2.3 ± 0.2	519.3 ± 77.6
NOCTH4	32.9 ± 0.5	15.9 ± 0.2	6.9 ± 0.1	0.9 ± 0.1
ACTB	17.0 ± 0.3			

d

Target gene	Ct							Slope	Amplification efficiency (%)
	HUVEC cDNA (no dilution)	1/4 dilution	$1/4^2$ dilution	$1/4^3$ dilution	$1/4^4$ dilution	$1/4^5$ dilution	$1/4^6$ dilution		
NOTCH1	18.4 ± 0.0	20.6 ± 0.1	22.5 ± 0.1	24.8 ± 0.2	26.5 ± 0.0	28.5 ± 0.1	30.6 ± 0.2	-3.35 ± 0.05	98.8
NOTCH2	21.0 ± 0.0	23.0 ± 0.0	24.9 ± 0.1	27.2 ± 0.1	29.2 ± 0.1	30.6 ± 0.3	33.8 ± 0.6	-3.43 ± 0.06	95.6
NOTCH3	27.0 ± 0.1	28.7 ± 0.2	31.0 ± 0.1	32.6 ± 0.1	34.8 ± 0.2			-3.36 ± 0.06	98.3
NOTCH4	21.9 ± 0.0	23.8 ± 0.0	25.8 ± 0.3	27.7 ± 0.2	29.9 ± 0.5	31.7 ± 0.1	34.3 ± 0.1	-3.37 ± 0.09	98.0

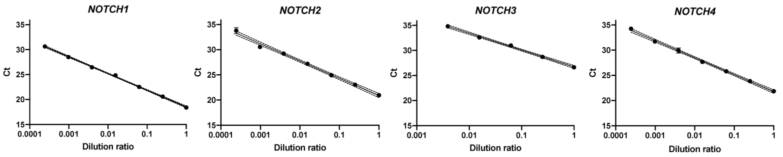

Supplementary Figure S2. Quantification of *NOTCH1-4* mRNA expression by RT-PCR and the assessment of PCR amplification efficiency.

(**a-c**) Quantitative PCR analyses of *NOTCH1-4* mRNAs in HUVECs (a), HMVECs-L (b) and HAoSMCs (c). Ct values and the relative mRNA abundance of each Notch paralogue against Notch1 are shown. *ACTB* (β-actin gene) was used as an internal control. ΔCt = (Ct value of *NOTCH1-4*) - (Ct value of *ACTB*). ΔΔCt = (ΔCt value of *NOTCH1-4*) - (ΔCt value of *NOTCH1*). ($2^{-\Delta\Delta Ct}$) x 100 (%) represents the relative mRNA abundance of each Notch paralogue against Notch1. (**d**) Determination of amplification efficiency of *NOTCH1-4* cDNAs. The cDNA from Dll4-stimulated HUVECs underwent serial 4-fold dilution and the diluted cDNAs were used as standard samples. Regression lines with 95% confidence intervals and amplification efficiency were determined as described in the Methods and shown as Table and Figures. Ct values and the slope of regression lines in Table are presented as means ± SEM from three independent experiments.

Supplementary Figure S3.

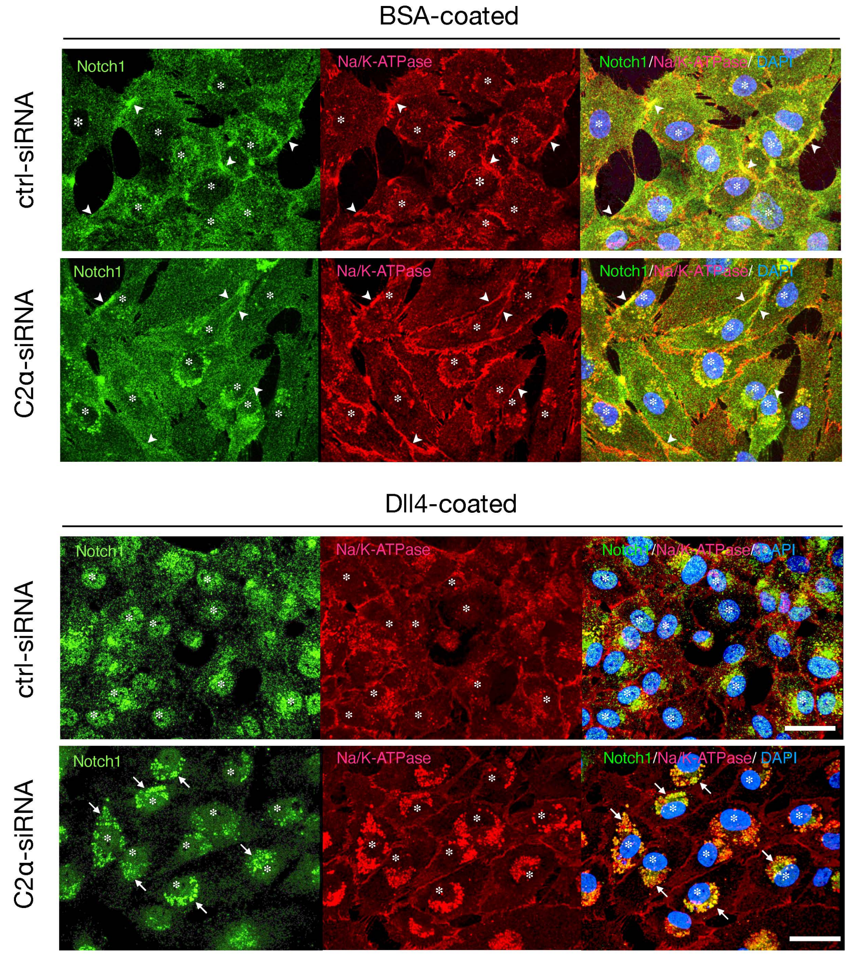

Supplementary Figure S3. Notch1 is translocated from plasma membrane to the perinuclear vesicles upon Dll4-stimulation.

PI3K-C2α-siRNA- or ctrl-siRNA-transfected HUVECs were incubated on BSA- or Dll4-coated dishes for 24 h. Cells were fixed and stained with anti-Notch1 and anti-Na/K-ATPase (plasma membrane marker) antibodies. Cells were observed by confocal fluorescent microscopy. In ctrl-siRNA-transfected HUVECs, intense Notch1 immunoreactivity was observed at the Na/K-ATPase-positive plasma membrane on BSA-coated dishes (white arrowheads in the upper panels) and Dll4 stimulation induced the disappearance of Notch1 immunoreactivity at the plasma membrane and its accumulation in the nuclei (asterisks). PI3K-C2α depletion inhibited Dll4-induced nuclear accumulation of Nocth1 and increased the perinuclear Notch1-positive dots (white arrows in the lower panel). In PI3K-C2α-depleted cells, Na/K-ATPase immunoreactivity was also accumulated at the perinuclear vesicles but it still existed at the cell boundary. Scale bars, 20 μm.

Supplementary Figure S4.

Fig. 1a

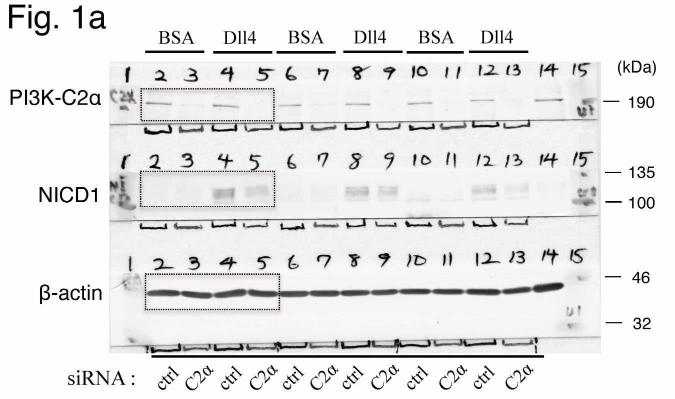

Fig. 2a

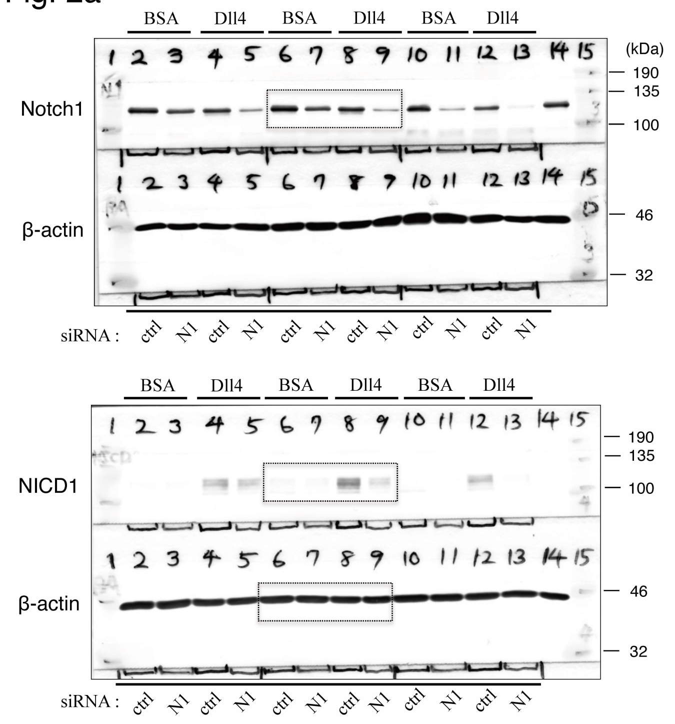

Fig. 2c

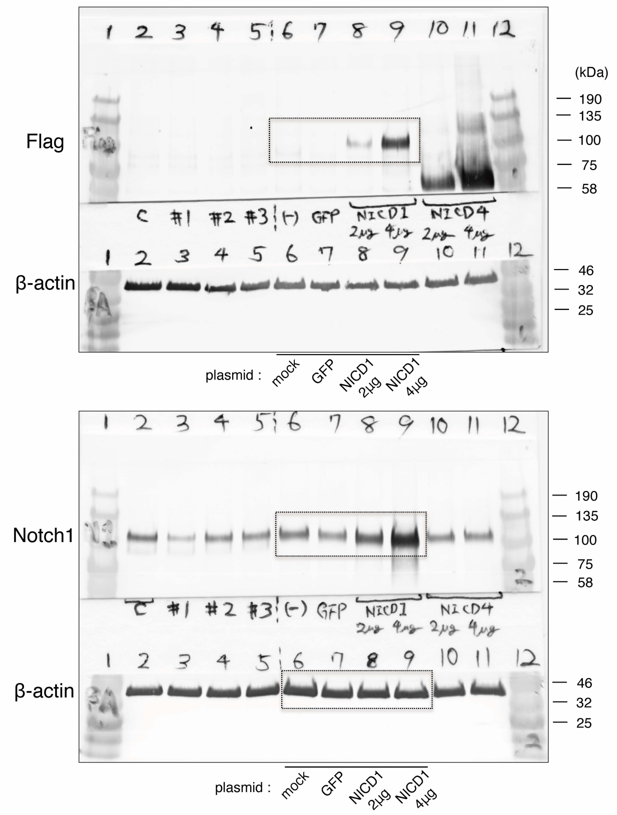

Supplementary Figure S4.

Fig. 3b

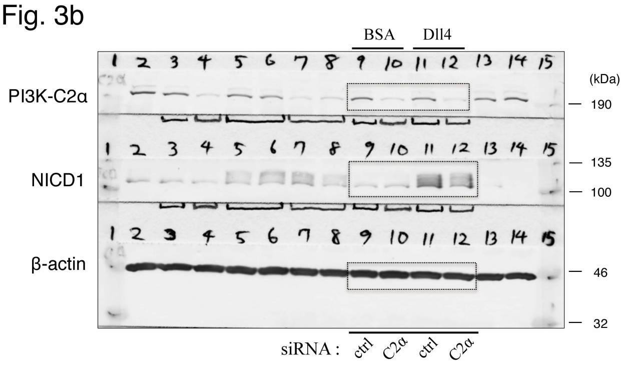

Fig. 5a

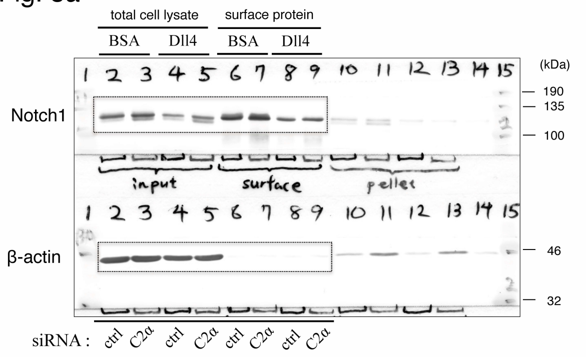

Fig. 3e

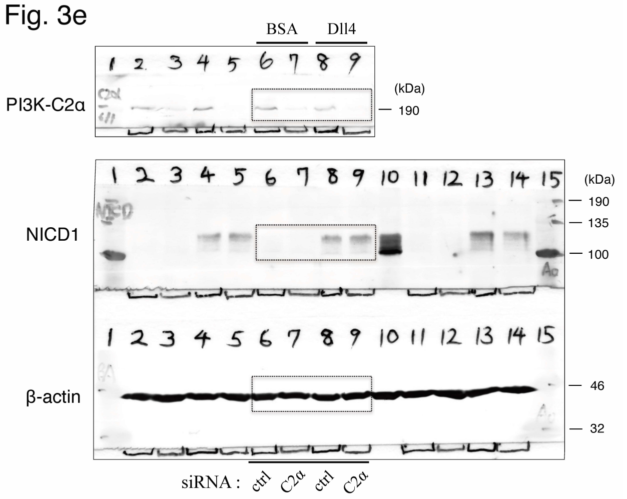

Fig. 5e

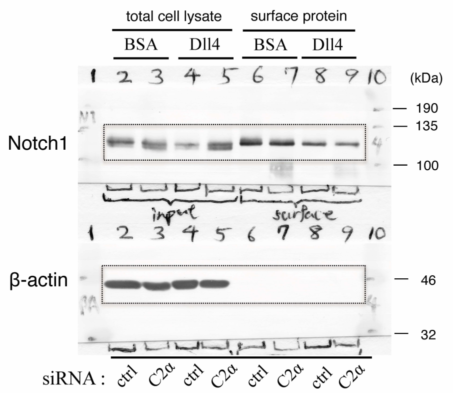

Supplementary Figure S4.

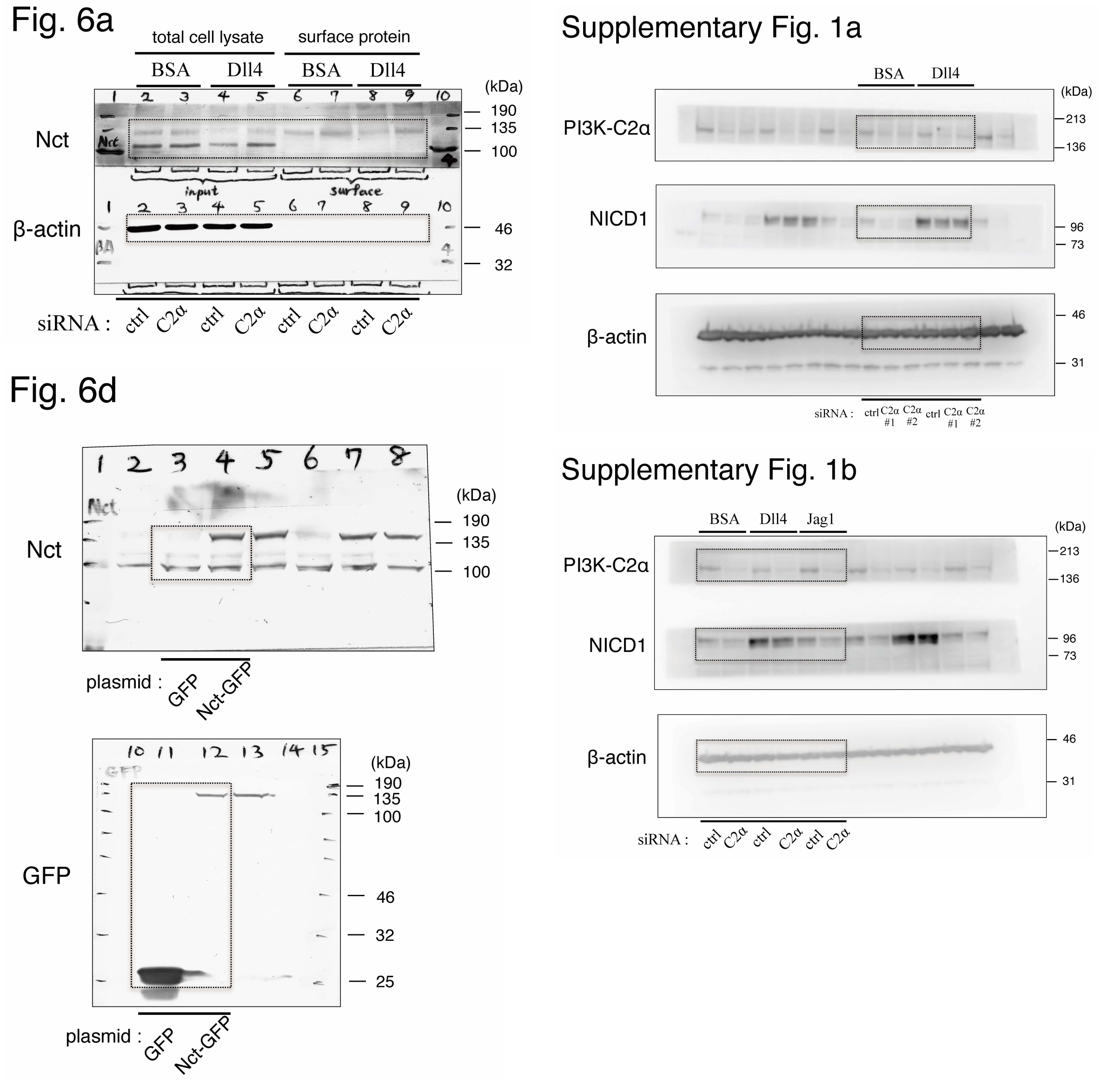

Supplementary Figure S4. Original images of Western blots used in Figures 1, 2, 3, 5 and 6, and Supplementary Figure S1.

The original membranes after the transfer of proteins were cut into two or three parts or non-cut, and then each part of the cut membrane pieces or the non-cut whole membranes were incubated with different primary antibodies as indicated. The dashed black boxes delineate the cropped regions presented in each Figure.
